# A benchmark for RNA-seq deconvolution analysis under dynamic testing environments

**DOI:** 10.1186/s13059-021-02290-6

**Published:** 2021-04-12

**Authors:** Haijing Jin, Zhandong Liu

**Affiliations:** 1 Graduate Program in Quantitative and Computational Biosciences, Baylor College of Medicine, Houston, USA; 2 Jan and Dan Duncan Neurological Research Institute at Texas Children’s Hospital, Houston, USA; 3 Department of Pediatrics, Baylor College of Medicine, Houston, USA

## Abstract

**Background:**

Deconvolution analyses have been widely used to track compositional alterations of cell types in gene expression data. Although a large number of novel methods have been developed, due to a lack of understanding of the effects of modeling assumptions and tuning parameters, it is challenging for researchers to select an optimal deconvolution method suitable for the targeted biological conditions.

**Results:**

To systematically reveal the pitfalls and challenges of deconvolution analyses, we investigate the impact of several technical and biological factors including simulation model, quantification unit, component number, weight matrix, and unknown content by constructing three benchmarking frameworks. These frameworks cover comparative analysis of 11 popular deconvolution methods under 1766 conditions.

**Conclusions:**

We provide new insights to researchers for future application, standardization, and development of deconvolution tools on RNA-seq data.

**Supplementary Information:**

The online version contains supplementary material available at 10.1186/s13059-021-02290-6.

## Introduction

Deconvolution refers to a process that separates a heterogeneous mixture signal into its constituent components. In the biomedical field, researchers have used deconvolution methods to derive cell type-specific signals [1–3] from heterogeneous mixture data. Cellular composition information is crucial for developing sophisticated diagnostic techniques because it enables researchers to track each cellular component’s contribution during disease progressions [4]. Although some experimental approaches like fluorescence-activated cell sorting (FACS), immunohistochemistry (IHC), and single-cell RNA-seq can derive cellular composition information [3], all these approaches are either restricted by their throughput or remain too costly and laborious for large-scale clinical applications. Deconvolution, which computationally decomposes mixture signals, provides a cost-effective way to derive cellular composition information and has the potential to bring considerable improvements in the speed and scale of relevant applications.

By January 2018, approximately 50 deconvolution methods had been developed [2]. While the speed of method development is increasing, researchers now face the new challenge of selecting appropriate methods for their analysis. In methodological papers, authors often use small benchmarks to illustrate the improvements of their methods. These benchmarks only contain a limited number of deconvolution methods and samples. Moreover, different research groups applied inconsistent testing frameworks with different simulation strategies, evaluation metrics, and cell type annotations, making it difficult for researchers to reach a solid conclusion on the method’s performance. Therefore, independent benchmarks are usually in need of rigorous and comprehensive comparisons [5]. Previously, Sturm et al. [3] and Cobos et al. [6] have generated independent benchmarks of reference-based and marker-based deconvolution methods on RNA-seq data. Focusing on spill-over effects, minimal detection fraction, and background predictions, Sturm et al. [3] suggested refining signature gene lists to improve deconvolution accuracy. On the other hand, Cobos et al. [6] focused on the impact of different normalization strategies, sequencing platforms of reference data, marker gene selection strategies, and missing cellular components in the reference. Compared with previous benchmarks, which mainly focused on the influence of reference profile and feature selection, our study focused on factors directly related to the mixture samples such as mixture noise level, quantification unit, cellular component number, weight matrix property, and unknown cellular contents. In addition to these factors, we also studied factors related to the testing framework construction, such as simulation model selection, evaluation metric selection, and measurement scale selection.

**Fig. 1:**
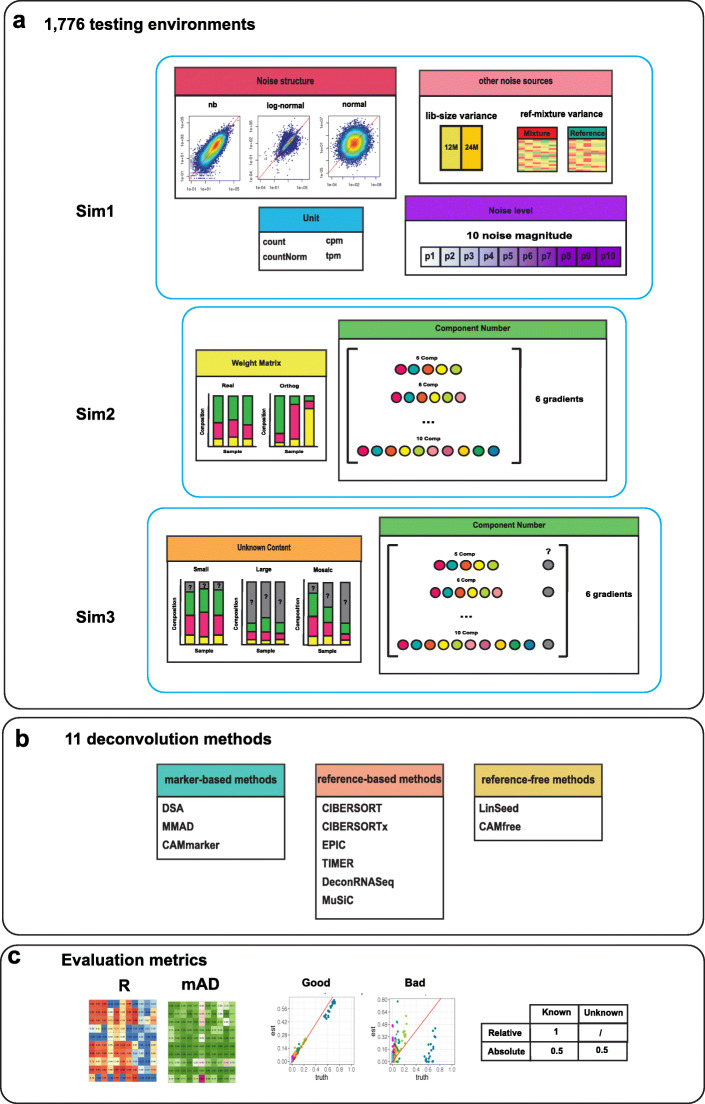
Overview of three in silico testing frameworks. **a** Three benchmarking frameworks were constructed to investigate the impact of seven factors that affect deconvolution analysis: noise level, noise structure, other noise sources, quantification unit, unknown content, component number, and weight matrix. **b** Eleven deconvolution methods are tested and have been categorized based on the required reference input: marker-based, reference-based, and reference-free. **c** Performance of the methods is assessed through Pearson’s correlation coefficient (*R*) and mean absolute deviance (mAD). Evaluation results are illustrated by heatmaps and scatter plots. When unknown content is involved, we derive evaluation metrics in both relative and absolute measurement scales

There are three types of benchmarking frameworks for the evaluation of deconvolution methods: in vivo framework [7], in vitro framework [8], and in silico framework [9, 10] (Additional file 2: Table S1). The in vivo testing framework mainly relies on indirect performance assessments and usually cannot derive a definite conclusion of the method’s performance. Only a few in vivo benchmarking datasets [3, 10] have coupled FACS results for direct performance assessments. Nevertheless, these benchmarking datasets only contain limited sample numbers and cannot provide a comprehensive performance assessment [3, 10]. The in vitro testing framework where mixtures are generated in the tube with predefined mixing compositions also suffers from limited sample numbers. Moreover, most benchmarks generated from the in vitro testing framework used “orthogonal” weights [8] during the mixing process, which would potentially result in over-optimistic conclusions. The in silico testing framework synthesizes heterogeneous mixture data by simulation. The primary goal of this study is to systematically investigate the impact of different biological and technical factors, where numerous finely tuned conditions need to be created [11]. A few biologically relevant cases cannot reveal the systematic biases since all technical and biological factors are confounded. Therefore, both in vivo and in vitro frameworks are not feasible for this type of systematic comparison due to the limitation in sample number and confounding factors. Careful consideration of these issues led us to select the in silico testing framework to systematically examine the impact of different biological and technical factors, which require large amounts of benchmarking datasets under controlled and finely tuned multi-factor testing environments (Fig. 1a and Table 1).

**Table 1:** Cellular components and datasets involved in three testing frameworks and variance analysis

Analysis	Cell types	Datasets
Variance analysis	CD8 T cells Whole blood Simulated mixtures (T, B, and mono)	GSE113590 GSE60424 GSE51984
Sim1_simModel	Simulated mixtures (T, B, and mono)	GSE60424 GSE51984 GSE64655
Sim1_libSize	Simulated mixtures (T, B, and mono)	
Sim2	6 gradients of cell types: Comp 5–10	GSE60424 GSE51984 GSE64655 GSE115736
Sim3	6 gradients of cell types: Comp 5–10 and one unknown component HCT116	GSE60424 GSE51984 GSE64655 GSE115736 GSE118490

Six gradients of cell types:

Comp 5—T, B, monocytes, neutrophils, and NK cells

Comp 6—T, B, monocytes, neutrophils, NK cells, and eosinophils

Comp 7—T, B, monocytes, neutrophils, NK cells, eosinophils, and myeloid DC

Comp 8—T, B, monocytes, neutrophils, NK cells, eosinophils, myeloid DC, and CD34+ HSC

Comp 9—CD4 T, CD8 T, B, monocytes, neutrophils, NK cells, eosinophils, myeloid DC, and CD34+ HSC

Comp 10—CD4 T, CD8 T, naive B, memory B, monocytes, neutrophils, NK cells, eosinophils, myeloid DC, and CD34+ HSC

To provide a reliable reference for the application and development of deconvolution methods, we compared 11 deconvolution methods (Fig. 1b) that cover three categories: marker-based, reference-based, and reference-free. To establish sophisticated benchmarking frameworks that mimic application scenarios of diverse biological systems, we designed three sets of benchmarking frameworks that simulated up to 1766 biological conditions with varying noise levels, library sizes, cellular component numbers, weight matrix properties, simulation models, and proportions of unknown contents (Fig. 1a and Additional file 2: Table S2). These simulated conditions will enable us to investigate the tipping point where each method deteriorates. To determine the impact of evaluation frameworks, we performed comparisons under different simulation models and measurement scales with two sets of evaluation metrics: correlation (Pearson’s correlation coefficient) and mean absolute deviation (mAD) (Fig. 1c, the “Methods” section). Moreover, we studied the impact of commonly applied simulation strategies, and by comparison to the real mixture data, we derived improved simulation strategies that can generate more complex and yet authentic in silico mixtures. Our results provide a dynamic testing landscape that allows the user to select the right method under the targeted experimental condition.

## Results

### Using simulation to generate diverse deconvolution testing environments

We designed three benchmarking frameworks to test the performance of deconvolution methods under multiple application scenarios. Each framework was designed to study the impact of specific technical and biological factors on deconvolution analysis (Fig. 1a and Table 1). The first benchmarking framework (Sim1) was designed to reveal the impact of the noise structure across dynamic noise levels (Additional file 1: Figure S1). The second benchmarking framework (Sim2) was designed to reveal the impact of the cellular component number and weight matrix property (Additional file 1: Figure S2a). The third benchmarking framework (Sim3) was designed to reveal the impact of unknown biological contents and measurement scales (Additional file 1: Figure S2b).

In an in silico benchmarking framework, a deconvolution testing environment consists of mixture data, reference data, ground truths, and deconvolution methods for testing. Mixture data refers to heterogeneous gene expression profiles for deconvolution. Reference data refers to homogeneous cell type-specific data that used to guide the deconvolution process. Ground truth refers to the real mixing proportions of constituent cell types in the mixture data. The accuracy of deconvolution methods can be assessed by comparing estimated proportions to the ground truths. Since reference data can vary based on the required input of the tested methods, we classified 11 deconvolution methods into the following categories: marker-based, reference-based, and reference-free (Fig. 1b and Additional file 2: Table S3). Marker-based methods such as DSA [12], MMAD [13], and CAMmarker [14] use marker gene lists to guide the deconvolution analysis. Reference-based methods such as CIBERSORT [9], CIBERSORTx [10], EPIC [15], TIMER [7], DeconRNASeq [16], and MuSiC [17] use cell type-specific gene expression profiles. Except for MuSiC [17], nearly all reference-based methods require signature gene lists as an additional input, which are differential expression genes across the cell types in the reference. MuSiC [17] applies weighted non-negative least squares regression (W-NNLS) and does not require any pre-determined gene sets. Finally, reference-free methods such as LinSeed [18] and CAMfree [14] do not require any external references in the deconvolution process. But these methods require reference profiles for the cluster annotation (assign cell types) after the deconvolution process.

### Selection of simulation model affecting deconvolution evaluation

The benchmarking framework Sim1_simModel is designed to learn the impact of noise structure across dynamic noise levels (Fig. 1a, Additional file 1: Figure S1a and Additional file 2: Table S2). To understand the impact of noise structures, we simulated noise based on three simulation models: normal, log-normal, and negative binomial (nb). These simulation models have been applied in the previous publications [9, 11, 16, 18, 19] to generate in silico mixtures. For each simulation model, we generated ten levels of noise to evaluate the robustness of deconvolution methods across dynamic noise levels (Additional file 2: Table S2 and the “Methods” section). To ensure the generality of our conclusion across different datasets and account for reference-mixture variance, we performed repeated mixture simulation with three independent blood datasets and created nine testing environments with different mixture-reference pairs (Additional file 2: Table S2 and the “Methods” section).

For the noise level, we observed that the accuracies of the deconvolution methods decreased as the noise level increased, which was exhibited as decreasing correlation (Additional file 1: Figure S3) and increasing mAD (Additional file 1: Figure S4) values. We also noticed that the impact of the RNA-seq quantification unit is trivial (Additional file 1: Figures S3 and S4) and thus selected the most commonly used unit tpm for the remaining illustrations of the results. Unless specifically indicated (as in Sim1_libSize and variance analysis), all results in this study are from mixture data with the tpm unit.

**Fig. 2:**
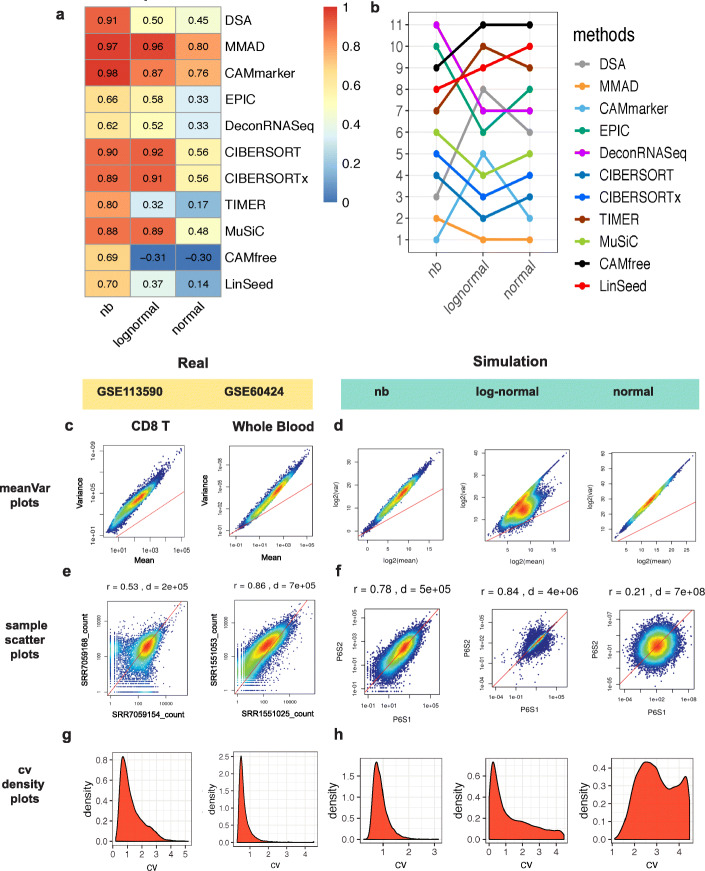
Evaluation results of Sim1_simModel and noise structure comparisons between real and simulated data. **a** Heatmap of the summarized evaluation results based on the Pearson’s correlation coefficients and **b** rankings of the tested deconvolution methods in the Sim1_simModel. In each heatmap, row indexes refer to the tested methods and column indexes refer to the simulation models (negative binomial, log-normal, and normal). **c**, **d** Mean-variance plots of **c** real and **d** simulated data. **e**, **f** Sample-sample scatter plots of **e** real and **f** simulated data. r, Spearman’s correlation coefficient; **d**, Euclidean distance. **g**, **h** Density plots of CV (coefficient of variation) of **g** real and **d** simulated data. Real data are derived from GSE113590 and GSE60424 (Additional file 1: Figures S6 and S7 contain detailed variance analysis results for each dataset). All simulated data in Fig. 2 are based on simulations derived from GSE51984 with the P6 noise level. Results in **a** and **b** are in the tpm unit; results in **c**–**h** are in count unit

To reveal the impact of the simulation models, we averaged the evaluation metrics across noise levels and generated summarized evaluation heatmaps (11 × 3) where 11 indicated the number of methods and 3 indicated the number of simulation models. Based on the summarized evaluation heatmaps of correlation (Fig. 2a) and mAD (Additional file 1: Figure S5a), we observed that the selection of the simulation model strongly affected the evaluation results. For instance, compared with evaluations from normal and log-normal groups, methods such as DSA [12], TIMER [7], and CAMfree’s [14] rankings were higher in the negative binomial group in both correlation (Fig. 2b) and mAD (Additional file 1: Figure S5b) metrics.

### The negative binomial model recapitulates noise structures of real data

In the Sim1_simModel, we found that the noise structure is the main factor obscuring deconvolution performance assessment (Fig. 2a, b, Additional file 1: Figure S5). To identify the simulation model that best recapitulates the essential characteristics of real data, we performed noise structure comparisons between real and simulated data by using mean-variance plots, sample-sample scatter plots, and coefficient of variance (CV) density plots (Fig. 2c–h). We selected two blood datasets that are sampled from dynamic biological conditions (different tissue and disease status) as representations of real datasets while using one blood dataset that is sampled from healthy donors as the simulation source (Table 1). Since comparing with simulation source, real data were sampled from more diverse biological conditions, we considered the noise observed in the real data as an upper boundary of the noise level.

We used the mean-variance plots to study the overall trend between variance and mean in both real and simulated data (P6 noise level) (Fig. 2c, d). As expected, we observed that the variance and the mean value of counts follow a linear trend in the log space with a clear overdispersion phenomenon, which is typical to the RNA-seq data [20] (Fig. 2c). However, in the simulation group, only the simulations generated from the negative binomial and normal models showed a similar mean-variance trend to the real data (Fig. 2d).

Next, we used sample-sample scatter plots to study the concordance trend of gene expression profiles (Fig. 2e, f). In the real data, we observed that lowly expressed genes exhibited larger relative deviances to the diagonal reference line (*y* = *x*) than highly expressed genes (Fig. 2e). This phenomenon indicates larger uncertainties in quantifying lowly expressed RNA molecules. In the simulation group, only the simulation data from the negative binomial model recapitulated higher deviances of lowly expressed genes (Fig. 2f).

We also compared the magnitude of noise between the real and simulated data. In the real data, the sample-sample Spearman’s correlation values ranged from 0.53 to 0.99 while the sample-sample Euclidean distances fluctuated around the order of 10^4^~10^5^ (Additional file 1: Figures S6 a and b and S7 a and b). Among the three tested simulation models, only the negative binomial model was capable of generating simulated profiles with comparable sample-sample correlation (0.57–0.98) and Euclidean distance (around the order of 10^4^~10^5^) to the real datasets (Additional file 1: Figure S8) while maintaining the mean-variance trend with the overdispersion phenomenon (Additional file 1: Figure S9).

We compared the density curve of coefficient variation (CV) values in the real and simulated data (Fig. 2g, h). The real data exhibited a unimodal bell-shaped curve, indicating that most of the genes had low to moderate levels of CV (Fig. 2g). In the simulation group, only simulations derived from the negative binomial model maintained the unimodal bell-shaped curve throughout all noise levels (Fig. 2h). CV density distributions of normal and log-normal models showed density curves that were skewed towards the high CV value from noise level P6 to P10, indicating unauthentic noise structure (Additional file 1: Figure S10b) in these simulations.

In conclusion, the negative binomial simulation model, which successfully recapitulated the mean-variance trend, sample-sample concordance, and the density of CV, presented the noise structure that was most similar to the noise structure in the real data. The negative binomial model also kept the magnitude of noise at comparable levels to the real data and thus should be considered as the most appropriate simulation model for generating in silico mixtures.

### Library size normalization is required to ensure the deconvolution accuracy

In Sim1_simModel, we observed the trivial impact of the quantification unit, but this might be due to the fact that all negative binomial simulated mixtures have the same library size (the “Methods” section). To further investigate the impact of quantification unit with varied library sizes, we designed Sim1_libSize (Additional file 2: Table S2 and Additional file 1: Figure S1b). In this framework, every simulated mixture set comprised two types of samples with 12 M and 24 M reads (the “Methods” section). For simplicity, we summarized the evaluation results across all 10 noise levels and generated evaluation heatmaps with dimensions 11 by 4 where 11 indicates the number of methods and 4 indicates the number of quantification units being tested.

**Fig. 3:**
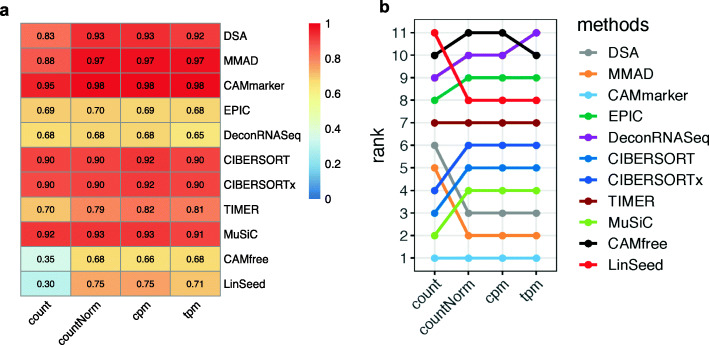
Evaluation results of Sim1_libSize. **a** Heatmap of the summarized evaluation results based on the Pearson’s correlation coefficients and **b** rankings of the tested deconvolution methods. In each heatmap, row indexes refer to the tested methods, and column indexes refer to the quantification units (count, countNorm, cpm, and tpm)

We observed that three methods, CIBERSORT [9], CIBERSORTx [10], and MuSiC [17], which implemented normalization procedures, showed satisfactory performance (*r* ≥ 0.9, mAD ≤ 0.1) regardless of the selected quantification unit (Fig. 3a, Additional file 1: Figure S11a). Six methods (DSA [12], MMAD [13], CAMmarker [14], TIMER [7], CAMfree [14], and LinSeed [18]) showed improved accuracy after library size normalization (Fig. 3a, Additional file 1: Figure S11a). We observed that, contrary to the Sim1_simModel (Additional file 1: Figure S3 and S4), the choice of quantification unit had a high impact on Sim1_libSize, which was shown by discrepant rankings of the tested methods (Fig. 3b and Additional file 1: Figure S11b). Because the only difference between these two benchmarking frameworks was the library size (the “Methods” section), we deduced that the inconsistent performance across different quantification units was due to the library size variation in the mixture dataset. Our results indicate using quantification units normalized by library sizes can mitigate the bias caused by library size variation (Fig. 3a and Additional file 1: Figure S11a). When a deconvolution method has no specific requirement on the quantification unit, we suggest researchers apply RNA-seq quantification units that are normalized by library sizes.

### Impact of cellular component number and weight matrix on deconvolution analysis

The weight matrix, which is defined by the cellular proportions across mixture samples, plays an important role in the deconvolution analysis as it directly affects the condition number (or orthogonality) of mixture datasets. While the weight matrix is not controllable in the analysis, researchers can roughly speculate weight matrix property based on previous cellular composition-relevant studies. Therefore, understanding the impact of weight matrix property will provide researchers new insights about method selection based on the mixture conditions.

To investigate the joint impact of the cellular component number and weight matrix property, we designed benchmarking framework Sim2 with six gradients of component number ranging from 5 to 10 and two types of weight matrices: “orthog” and “real” (Fig. 1a, Additional file 2: Table S2 and Additional file 1: Figure S2a). The “orthog” weight matrix was generated by minimizing the condition number, and the “real” weight matrix was constructed based on whole blood immune cell proportions in the real biological samples [21] (the “Methods” section). We discarded the CAMfree [14] method in Sim2 due to the poor scalability of CAMfree [14] on mixtures with large component numbers.

**Fig. 4:**
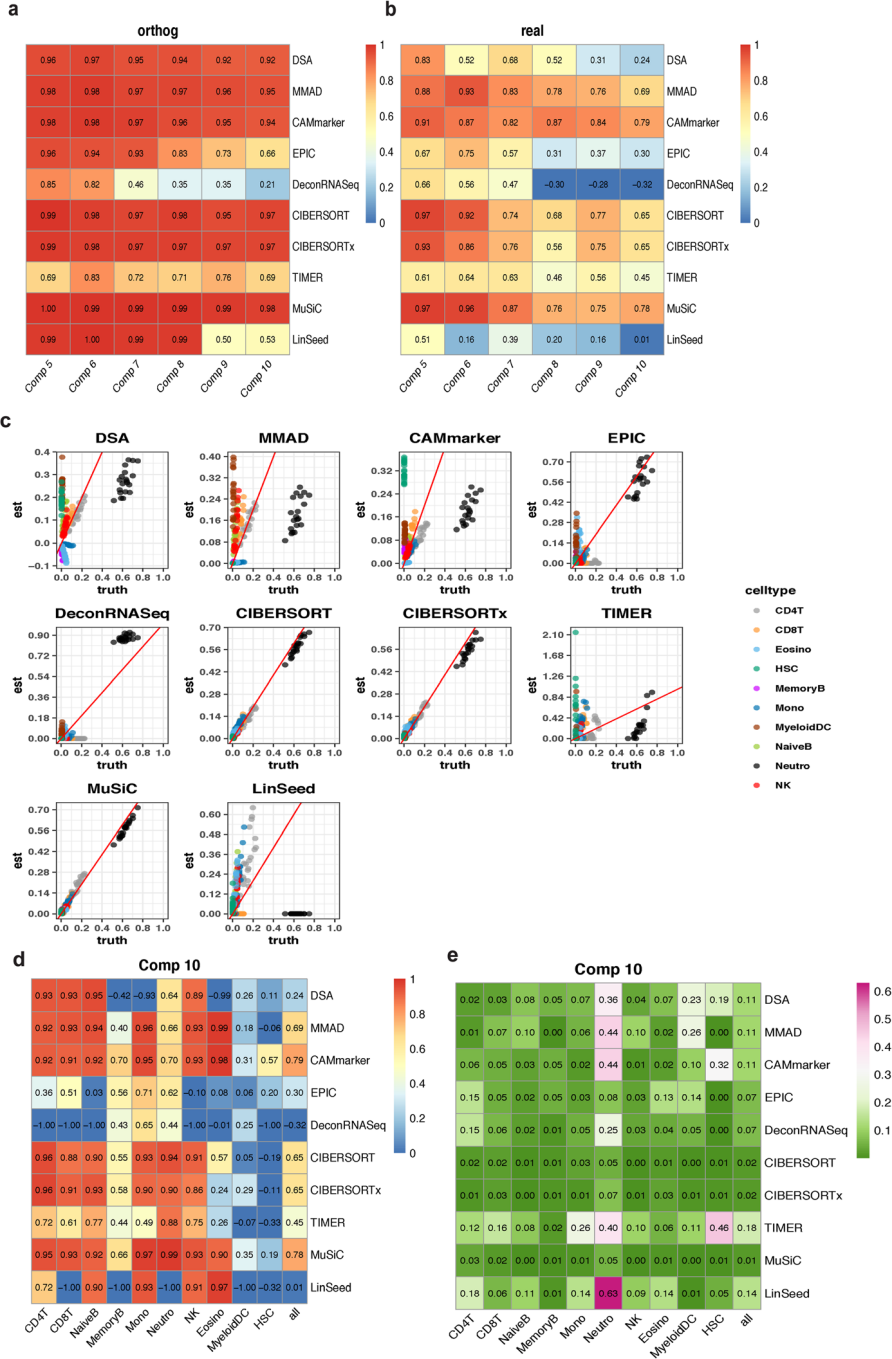
Evaluation results of Sim2. **a**, **b** Heatmaps of the summarized evaluation results based on the Pearson’s correlation coefficients with **a** “orthog” weight matrix and **b** real weight matrix. In each heatmap, row indexes refer to the tested methods, and column indexes refer to the cellular component numbers. **c** Scatter plots of estimated weights vs. ground truths of “real” mixtures with 10 cellular components. **d**, **e** Cell type-specific evaluation results of “real” mixtures consist of 10 cellular components based on **d** Pearson’s correlation coefficient and **e** mean absolute deviance. In each heatmap, row indexes refer to the tested methods, column indexes refer to the cell types, and the last column “all” refers to the averaged evaluation results across all cell types

We found that nearly all deconvolution methods achieved higher levels of accuracies with the “orthog” weight matrices (Fig. 4a) relative to the “real” weight matrices (Fig. 4b), indicating that the mathematical property of the weight matrix had a significant impact on deconvolution analysis. In the mixtures with five components (Comp 5), eight methods (DSA [12], MMAD [13], CAMmarker [14], EPIC [15], CIBERSORT [9], CIBERSORTx [10], MuSiC [17], and LinSeed [18]) exhibited high accuracy levels(*r* ≥ 0.95, mAD ≤ 0.05) in the “orthog” group (Fig. 4a and Additional file 1: Figure S12a), while only two of those eight methods (CIBERSORT [9] and MuSiC [17]) in the “real” group achieved the same level of accuracy (Fig. 4b and Additional file 1: Figure S12b).

In addition to the impact of the weight matrix selection, the cellular component number also affected the deconvolution accuracy. In both the “orthog” and “real” groups, the majority of the methods exhibited poorer performance as the cellular component number increased (Fig. 4a,b and Additional file 1: Figure S12). It is also worth noting that none of the tested deconvolution methods showed a correlation higher than 0.9 with mixtures consist of large cellular component numbers (Comp 7 to Comp 10) in the “real” group (Fig. 4b).

To further investigate the performance of deconvolution methods with large component numbers, we explored the accuracies of mixtures with 10 cellular components and the “real” weight matrix by drawing scatter plots of the estimated proportions and ground truths (Fig. 4c, data corresponds to the last column of Fig. 4b and Additional file 1: Figure S12b). Unexpectedly, we found that the correlation evaluation metric, which was considered as the golden standard for the evaluation of deconvolution methods, cannot reflect the deviance of estimations from ground truths (Fig. 4c). However, the deviance of estimation can be shown by another evaluation metric mAD (Additional file 1: Figure S12). For instance, MMAD [13] and CAMmarker [14] performed relatively well on the correlation evaluation metric (*r* ≥ 0.65, Fig. 4b), but both methods had mAD values larger than 0.1, indicating the existence of large estimation deviance (Additional file 1: Figure S12b). By combining information from two evaluation metrics (Fig. 4b and Additional file 1: Figure S12b), we reached the same conclusion shown by the scatter plots (Fig. 4c) that the best performers were CIBERSORT [9], CIBERSORTx [10], and MuSiC [17]. All three methods achieved high accuracies on both correlation evaluation metric (*r* ≥ 0.65) (Fig. 4b) and mAD evaluation metric (mAD ≤ 0.02) (Additional file 1: Figure S12b) in the Comp 10 mixture with “real” weight matrix.

To understand the impact of each cellular component on deconvolution analysis, we drew evaluation heatmaps with cell type-specific correlation and mAD values (Additional file 1: Figures S13 and S14). Based on the evaluation heatmap of mixtures with ten cellular components and the “real” weight matrix, which is the most complicated in silico mixture set in the Sim2 benchmark framework, we identified three best performers: CIBERSORT [9], CIBEERSORTx [10], and MuSiC [17]. Firstly, we found that all three methods correctly estimated major cellular components (*r* ≥ 0.85, mAD ≤ 0.05), such as neutrophils, CD4 T, and CD8 T in the mixtures (Fig. 4d and e). Secondly, while all three methods failed to estimate the linear trend of proportions of rare cell subpopulations (*r* − 0.19~0.35) that occupied less than 1% in the mixture, such as myeloid DC, and hematopoietic stem cells (HSC), the three methods correctly identified them as minor components and did not attribute the percentages of other cell types to these rare cell populations (mAD 0~0.01) (Fig. 4d, e). Finally, we discovered that marker gene-based methods DSA [12], MMAD [13], and CAMmarker [14] and reference-free method LinSeed [18] showed high mAD values on neutrophil proportion estimation indicating larger deviances of their estimations on the major components (mAD 0.36~0.63) (Fig. 4e).

To understand which cellular component caused the performance deterioration, we inspected the cell type-specific evaluation results of the “real” weight matrices across the six component gradients (Additional file 1: Figure S13). We found that the rare cellular component myeloid DC (~ 0.3–0.9%) in the Comp 7 mixture has the lowest correlation to the true proportions, which might be due to its high similarity in gene expression to the monocytes [22]. However, introducing a relatively distinct HSC in the Comp 8 mixture further exacerbated the performance deterioration (Additional file 1: Figures S13 and S14, “real” group). Therefore, we concluded that the deterioration of deconvolution performance on mixtures with large component number is due to the confounding effect of both the highly correlated cellular component and the rare cellular component in the mixture dataset.

To further explore the impact of minor components and highly correlated proportions, we synthesized mixtures with two types of weight matrices: “dominant” and “uniform” (the “Methods” section). In the “dominant” group, there is one major cellular component (T cell or CD4 T cell) and K-1 minor components (Additional file 1: Figure S15a). In the “uniform” group, all cellular components have similar levels of proportions (Additional file 1: Figure S15b). Consistent with previous analysis on “real” and “orthog” weight matrices, we found that CIBERSORT [9], CIBERSORTx [10], and MuSiC [17] showed satisfactory accuracy (Additional file 1: Figure S15) in these two simulations, which indicates the high robustness of these three methods to the weight matrix property.

### Impact of the unknown component on deconvolution analysis

Unknown biological content is another major factor that influences deconvolution analysis for several reasons. First, unknown content could be treated as a source of noise unless explicitly modeled by deconvolution methods [9, 15]. Second, unknown content is not counted in the estimated cell type proportions and violates the sum-to-one assumption applied by the majority of deconvolution methods [2, 8].

To study the impact of unknown biological content on deconvolution analysis, we designed a benchmarking framework that contains mixtures with three sets of tumor spike-ins: the “small” group refers to mixtures with low levels of tumor spike-ins (20–30%), the “large” group refers to mixtures with high levels of tumor spike-ins (70–80%), and the “mosaic” group refers to mixtures with dynamic levels of tumor spike-ins (5–95%). Tumor spike-ins were introduced to the 12 mixture sets generated in the Sim2 framework to analyze the joint impact of the component numbers, weight matrix properties, and unknown biological contents (Fig. 1a, Additional file 2: Table S2 and Additional file 1: Figure S2b). At the performance assessment step, we used two sets of ground truths to derive evaluation results that represent different measurement scales (Additional file 2: Table S4 and the “Methods” section). The first set of ground truths used the absolute proportions of immune cell types and led to “absolute” deconvolution accuracy. The second set of ground truths used the relative proportions of immune cells and led to “relative” deconvolution accuracy. We evaluated ten methods and two additional specific method settings TIMERtumor [7] and EPICabsolute [15], which are tailored for deconvolution analysis with unknown tumor contents (Additional file 2: Table S3).

**Fig. 5:**
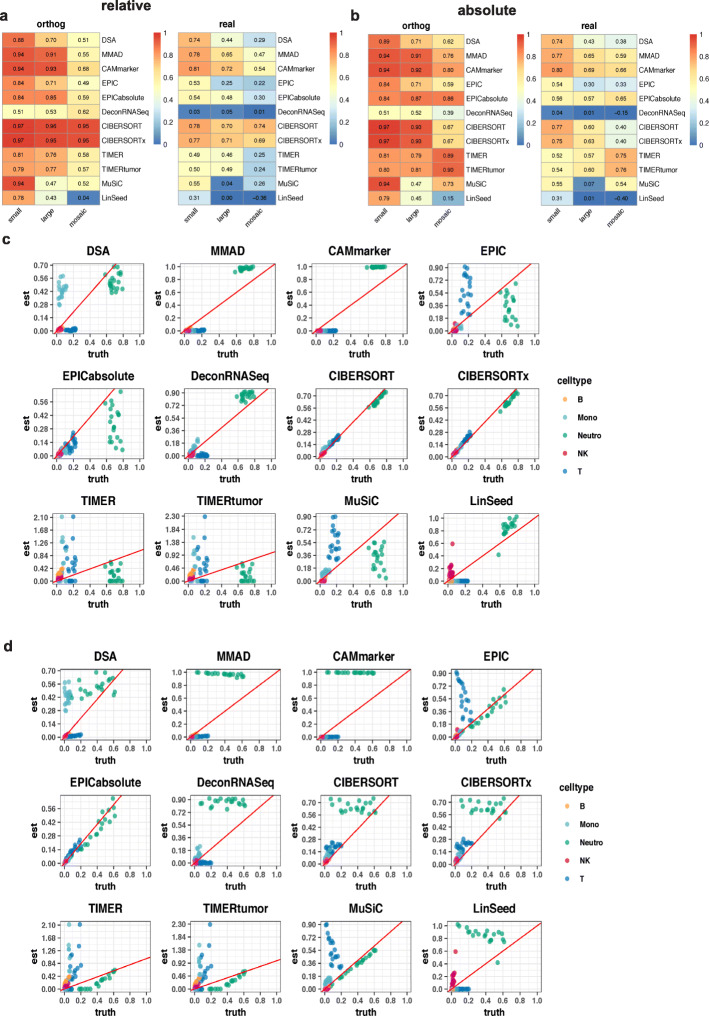
Evaluation results of Sim3. **a**, **b** Heatmaps of the summarized evaluation results based on the Pearson’s correlation coefficients on the **a** relative measurement scale and **b** absolute measurement scale. In each heatmap, row indexes refer to the tested methods, and column indexes refer to the types of tumor spike-ins (small, large, and mosaic). **c**, **d** Scatter plots of the estimated weights vs. ground truths of mixtures consist of 5 cellular components and mosaic tumor spike-ins. **c** Estimated weights vs. relative ground truth. **d** Estimated weights vs. absolute ground truth

Our results indicated that the weight matrix property was the leading factor that affected the deconvolution accuracy with the “orthog” group presented higher accuracies in most deconvolution methods across all tumor content groups (Fig. 5a, b and Additional file 1: Figure S16). In addition to the weight matrix property, we found that the size of tumor content also affected the deconvolution accuracy as we observed deconvolution methods performed better on mixtures with smaller tumor content (Fig. 5a, b and Additional file 1: Figure S16). Moreover, we found that when evaluated on different measurement scales (Fig. 5a, b and Additional file 1: Figure S16), most of the methods showed inconsistent performances. For example, CIBERSORT [9] and CIBERSORTx [10] showed greater accuracies (*r* 0.69~0.97, mAD 0.02 – 0.04) on the relative measurement scale (Fig. 5a and Additional file 1: Figure S16a) than on the absolute measurement scale (*r* 0.4~0.97, mAD 0.04~0.11) (Fig. 5b and Additional file 1: Figure S16b). Meanwhile, EPICabsolute [15] showed greater accuracies on the absolute measurement scale (*r* 0.56~0.87, mAD 0.02~0.05) (Fig. 5b and Additional file 1: Figure S16b) than on the relative measurement scale (*r* 0.3~0.85, mAD 0.06~0.12) (Fig. 5a and Additional file 1: Figure S16a).

To further investigate the performance of deconvolution methods under the cell type resolution, we drew scatter plots of the estimations from 5 Comp mixtures with “mosaic” tumor spike-ins and “real” weight matrix (Fig. 5c, d). On the relative measurement scale, CIBERSORT [9] and CIBERSORTx [10] were the top performers and achieved high accuracy (*r* ≥ 0.95, mAD ≤ 0.05) (Fig. 5c and Additional file 1: Figure S17 first column in the “mosaic” and “real” groups). However, on the absolute measurement scale, EPICabsolute [15] was the top performer and correctly estimated the absolute immune cell proportions (*r* ≥ 0.95, mAD ≤ 0.05) (Fig. 5d and Additional file 1: Figure S18 first column in the “mosaic” and “real” groups).

Next, we checked the robustness of the three best performers in terms of component number and tumor content in the “real” weight matrix group. The robustness of CIBERSORT [9] and CIBERSORTx’s [10] performance to the component number is high ( *r* 0.45~0.95, mAD 0.02~0.05) on the relative measurement scale (Additional file 1: Figure S17, the “real” group). EPICabsolute [15] also showed good robustness to the component number except for Comp 10 mixture (*r* 0.43~0.95, mAD 0.01~0.07, results of Comp 10 is excluded) on the absolute measurement scale (Additional file 1: Figure S18, the “real” group). We also found that having a larger variance in tumor content increased the accuracy of EPICabsolute [15], as we observed that with mosaic tumor spike-ins, EPICabsolute achieved greater accuracies (*r* 0.31~0.95, mAD 0.02~0.05) than other tumor spike-in groups (*r* 0.17~0.84, mAD 0.01~0.07) (Additional file 1: Figure S18, the “real” group) on the absolute scale. Consistent with the results in Sim2, we observed decreasing accuracies of CIBERSORT [9], CIBERSORTx [10], and EPICabsolute [15] with the increasing component number (Additional file 1: Figure S17a and Additional file 1: Figure S18a). We reasoned that this phenomenon was due to the difficulty of current deconvolution methods estimating rare subpopulations and closely related cell types.

Our results revealed the impact of unknown biological content on deconvolution analysis. We found that both the size (large vs. small spike-ins) and variance (large vs. mosaic spike-ins) of unknown content affected the deconvolution analysis. We also observed a discrepancy in performance evaluation when using different measurement scales. When the relative scale was used, CIBERSORT [9] and CIBERSORTx [10] were the top performers. When the absolute scale was used, EPICabsolute [15] was the top performer.

## Discussion

In this study, we designed three in silico benchmarking frameworks to systematically investigate the impact of several biological and technical factors. We identified top-performing deconvolution methods for each framework and illustrated the strengths and weaknesses of the tested methods under different application scenarios. Additionally, we provided strategies for mitigating systematic biases caused by different technical and biological factors such as varied library sizes, simulation models, and cellular compositions.

In the first framework (Sim1), we explored the impact of noise structure at different noise levels. We identified CAMmarker [14], MMAD [13], DSA [12], CIBERSORT [9], CIBERSORTx [10], and MuSiC [17] as the best performers since these methods showed high levels of accuracy at varying noise levels. For the noise structure, we identified the negative binomial as the best simulation model that captured the essential characteristics of the real data. In the second framework (Sim2), we explored the impact of the cellular component number and the weight matrix property. We identified CIBERSORT [9], CIBERSORTx [10], and MuSiC [17] as the top performers since these three methods achieved a high degree of accuracy across a gradient of cellular component numbers with both “orthog” and “real” weight matrices. We also found that all marker gene-based methods exhibited larger estimation deviances from ground truths. This type of estimation biases was shown in the scatter plots and can be quantitatively captured by the mAD evaluation metric, indicating the necessity of using mAD as a supplementary evaluation metric for deconvolution performance assessment. In the third framework (Sim3), we explored the impact of unknown biological content and measurement scales. On the relative measurement scale, CIBERSORT [9] and CIBERSORTx [10] were the best performers. On the absolute measurement scale, EPICabsolute [15] was the best performer. Our analysis also showed different evaluation results under the absolute and relative measurement scales. To our best knowledge, no previous deconvolution benchmark has documented this phenomenon.

Based on the results obtained in the present investigation, we offer suggestions for best practices of deconvolution analysis and evaluations. For the benchmarking data generation, we recommended that researchers (1) use the negative binomial model as the primary simulation model for in silico mixture data generation; (2) reference real biological composition data when building weight matrices,; (3) consider at least two evaluation metrics: one is used for checking linear concordance between estimation and ground truth, and the other one is used for checking estimation deviances; (4) when unknown biological content is involved, beware of the influence caused by different measurement scales (absolute vs. relative); and (5) construct multi-factor conditions on a large scale to ensure the robustness and comprehensiveness of the benchmark.

For deconvolution analysis, we suggest that researchers (1) use the quantification unit (countNorm, cpm, or tpm) that is normalized by library sizes; (2) check for the compositional information from previous publications. When the targeted tissue type has a relatively stable composition over several samples, consider using deconvolution methods that are robust to non-orthogonal weight matrices such as CIBERSORT [9], CIBERSORTx [10], and MuSiC [17]. When an unknown cellular component is expected (i.e., tumor sample) and the researcher needs to derive absolute proportion, consider using methods like EPIC [15], which is specifically tailored for deconvolution with unknown content; and (3) when referencing benchmark paper to select the optimal method, beware of different technical factors that may derive different estimation accuracies such as the resolution of analysis (number of cellular components), the variance of proportions across samples (weight matrix property), reference selection, evaluation metric selection, and measurement scale selection.

In addition to the suggestions listed above, previous benchmark publications also clarified the impact of signature matrices [1], multicollinearity issue [9], spill-over effects [3, 23], minimal detection fraction [3], background predictions [3], marker/signature gene selection [3], and the variance between reference and mixture sources [4]. Some deconvolution methods can derive both cell type-specific expression and composition signals [2]. Besides benchmarks focusing on cellular composition information, more benchmarks that derive accuracies of estimating cell type-specific expression are still needed. In this study, we used customized references that only contain cell types in the mixture source. However, previous studies have shown the impact of reference selection on deconvolution analysis [4]. Therefore, the joint impact of reference selection and mixture sample properties still need to be investigated.

For the future advancement of deconvolution analysis on RNA-seq data, we suggest that more efforts be put into the refinement of simulation models to generate more authentic in silico testing environments that mimic diverse application scenarios. In this study, the weight matrix property was revealed as the most important factor affecting deconvolution analysis, which was overlooked in prior research. Therefore, more studies on the cellular compositional information and its corresponding effects on deconvolution analysis are needed. All these improvements in simulation and benchmarking strategies will further enhance the efficiency of deconvolution method development.

## Methods

### Data processing

Raw SRA files were downloaded from the GEO repository, processed by SRA Toolkit (2.10.0) [24]; reads were aligned to the human reference GRCh38 (v95, hard masked) using alignment tool STAR (2.6.1) [25]; and quantification was performed using RSEM (1.3.1) [26] with default parameter settings. Quantification matrices with the count, tpm, and fpkm units were loaded into R (3.6.1) [27] for feature ID transformation, duplication removal, and low-abundant gene removal. For low-abundant gene removal, we relied on two parameters: minimum sample threshold (GSE113590 [28]—4; other datasets—5) and minimum expression threshold (10 counts, 1 tpm, and 1fpkm). For instance, the filtering parameter (5, 10) is used to retain genes with more than 10 counts in at least 5 samples. GSE113590 only has 4 samples per cellular category, and we set the minimum sample threshold as 4. In the Sim1, we performed filtering independently on each dataset with a minimum sample threshold set at 5. For Sim2 and Sim3, we first concatenated samples into one matrix and then performed filtering with a minimum sample threshold set at 10. For the information of datasets involved in Sim1, Sim2, and Sim3, please refer to Table 1.

### Marker gene selection

For the marker gene selection, we selected genes that are highly expressed in the targeted cell type and lowly expressed in other cell types. The expression threshold is set at the 80th percentile for high expression (the targeted group) and 50th percentile for low expression (other groups). To successfully derive marker genes with a larger number of cellular components, we gradually relaxed the criteria by decreasing parameter *p*, which is the percentage of samples that pass the criteria (initial value of *p* = 0.95) by a step parameter (default value *s* = 0.03) until there are at least two marker genes determined.

### Signature gene selection

We performed differential expression testing on all cell type pairs using DESeq2 [29]. We selected genes with *p*_adj_ ≤ 0.01 and ∣ log 2FoldChange ∣  ≥ 10.

### Reference generation

The reference profiles for deconvolution analysis were constructed from purified homogeneous samples. For Sim1 and Sim2, we used a total of ten cell types listed in Table 1. For instance, RNA-seq profiles of purified T cells, B cells, and monocytes were used as references for deconvolution methods in Sim1. For Sim3, we used up to ten immune components in the reference and excluded the unknown component (HCT 116) in the reference.

### Benchmarking framework construction

Three benchmarking frameworks are constructed to study the impact of different technical and biological factors on deconvolution analysis (Fig. 1, Table 1, and Additional file 2: Table S2). We created simulated mixture data *M* (*N* by *J*) by multiplying component source profiles *S* (*N* by *K*) to the predefined weight matrix *W* (*K* by *J*). Here, *N* is the number of genes, *J* is the number of samples, and *K* is the number of cellular components. The noise term *ε* is used to model sample-to-sample variability where the value of *ε* determines the noise level. All simulated mixtures were based on purified homogeneous profiles, and only samples derived from homogeneous sources were picked into the component source profiles. We avoided using heterogeneous samples identified as “whole blood,” “whole white blood,” or “pbmc,” for the source profile generation. Those heterogeneous samples are only used in the variance analysis (Fig. 2c, e, g) to deduce the upper boundary of the noise in the real data.

$$ M=S\times W+\varepsilon $$

#### Sim1

In Sim1, we aimed at understanding the impact of noise from different aspects such as noise structure and noise level (Additional file 1: Figure S1). Sim1 consists of two sub frameworks: Sim1_simModel and Sim1_libSize, where Sim1_simModel focuses on the noise structure, and Sim1_libSize focuses on the noise caused by varied library sizes. We used GSE60424 [30], GSE64655 [31], and GSE51984 [32] data to generate Sim1. 

#### Sim1_simModel

In this benchmarking framework, we mainly focused on the impact of the simulation model that was used to generate noise in the in silico mixtures. We selected three models for this study, which are the normal, log-normal, and negative binomial models. For each simulation model, we generated ten gradients of noise, and the noise level is controlled by a corresponding variance parameter. In Sim1_simModel, the library size of negative binomial model-based simulations was set at 12 M.

Normal model:

$$ M={2}^{\log_2\left(S\times W+1\right)+N\left(0,\sigma {p}_t\right)} $$

Log-normal model:

$$ M=S\times W+{2}^{N\left(0,\sigma {p}_t\right)} $$

In both log-normal and normal simulation models, the level of noise is controlled by the product of a constant parameter *σ* and a perturbation level parameter *p*_*t*_. In this study, we set *σ* to 10 based on previous publications [9] and set *p*_*t*_ as an element of a length-10 set {0, 0.1, 0.2, …, 0.9}. By tuning the *p*_*t*_ value, we were able to generate mixtures with 10 levels of noise.

Negative binomial model:

$$ {\mu}_{i0}={r}_{i0}\times {L}_j $$

$$ {\mu}_{ij}=\mathrm{Gamma}\left(\mathrm{shape}=\frac{1}{\sigma_i^2},\mathrm{scale}=\frac{\mu_{i0}}{\mathrm{shape}}\right) $$

$$ {\sigma}_i=\left(1.8\times {p}_t+\frac{1}{\sqrt{\mu_{i0}}}\right)\times \exp \left(\frac{\delta }{2}\right)\mathrm{where}\delta \sim N\left(0,0.25\right) $$

$$ {v}_{ij}=\mathrm{Poisson}\left({\mu}_{ij}\right) $$

We followed the simulation process suggested by Law et al. [19] and used *p*_*t*_ to control the noise level for simulation. All variables are scalars unless specifically indicated. *r*_*i*0_ is the expected genomic feature proportion of gene *i* in a cellular component. *L*_*j*_ is the library size of sample *j*, and *μ*_*i*0_ is the expected gene expression in the simulation. In the negative binomial model, two layers of variance are added from the gamma distribution and poisson distribution. We derived gene expression value *μ*_*ij*_ from gamma sampling to model the biological variance. In the gamma distribution, the variance is determined by the shape parameter *σ*_*i*_. We used *p*_*t*_, an element in a length 10 set {0.1, 0.2, … , 0.9, 1}, to regulate the value of *σ*_*i*_ to control the noise level in the negative binomial simulation. Then, we performed poisson sampling to model technical variance and get the final simulated expression value *v*_*ij*_.

### Reference mixture variance

To ensure the universality of our conclusion on different datasets, we applied the Sim1 framework on 3 blood datasets to generate reference and in silico mixtures (Additional file 1: Figure S1). Different from previous studies that concatenate samples derived from different datasets, we generated 3 sets of simulated mixtures and 3 sets of references independently and then used combinations of mixtures and references to generate 9 replicated testing environments for each noise level. For one testing environment, there are 9 (3 times 3) deconvolution results, and 6 of them have mixture-reference pairs derived from different data sources. For simplicity, we only presented the averaged performance across 9 mixture-reference pairs, but the impact of mixture-reference variance is considered in this analysis. The abovementioned mixture-reference variance modeled in Sim1 is named as other noise sources in Additional file 2: Table S2.

### Quantification units

To understand the impact of quantification units over different application scenarios, we generated simulations of the most commonly used RNA-seq quantification units: count, countNorm, cpm, and tpm. For a single sample, the unit transformation is as follows:

$$ {\mathrm{cpm}}_i=\frac{{\mathrm{Count}}_i}{\sum \limits_{i^{\prime }}C{\mathrm{ount}}_{i^{\prime }}}\times {10}^6 $$

$$ {\mathrm{countNorm}}_i=\frac{{\mathrm{Count}}_i}{\sum \limits_{i^{\prime }}{\mathrm{Count}}_{i^{\prime }}}\times {L}_{\mathrm{Median}} $$

$$ {\mathrm{tpm}}_i=\frac{{\mathrm{Count}}_i}{L_i}\times \left(\frac{1}{\sum \limits_{i^{\prime }}\frac{{\mathrm{Count}}_{i^{\prime }}}{L_{i^{\prime }}}}\right)\times {10}^6 $$

Here, *i* is the index of the targeted gene, *L*_Median_ is the median library size, and *i*′ refers to any gene in the profile. cpm and countNorm are normalized by library size. tpm is normalized by both library size and feature length.

#### Sim1_libSize

In this testing framework, we mainly focused on bias derived from varied library sizes (Table 1 and Additional file 1: Figure S1b). We first simulated mixtures based on the negative binomial model with the same noise gradient in Sim1_simModel. The library size is controlled by the library size parameter *L*_*j*_ in the negative binomial model. For every simulation dataset that consists of 20 simulated profiles, we set the library size of the first ten samples as 12 million reads and the remaining ten samples as 24 million reads.

#### Sim2

In this benchmarking framework, we studied the impact of the cellular component number and the mathematical property of the weight matrix (Table 1 and Additional file 1: Figure S2a). Mixtures are generated based on the negative binomial model with the noise close to *p*_1_ level. For component number, we generated six sets of mixtures constructed from 5 components up to 10 components. The cellular components used in each gradient and the raw data source for Sim2 are listed in Table 1. For the weight matrix, we generated 4 types of weight matrices: “orthog,” “real,” “dominant,” and “uniform.” We used GSE60424 [30], GSE64655 [31], GSE51984 [32], and GSE115736 [33] data to generate Sim2. 

### Weight simulations

For the weight simulation, we mainly relied on random sampling from the uniform distribution. We make use of different sets of range parameters (min and max) to generate four types of weight matrices: orthog, real, dominant, and uniform. After the initial sampling, all weights will be rescaled so that the weights of all components sum to 1.

“Orthog” refers to the idealized weight matrix with a small condition number, which provides a relatively optimal mathematical condition for deconvolution analysis. We first simulated 1000 matrices (*K* by *J*) by randomly sampling weights from a uniform distribution and then rescaled sampled weights so that the weights from all cellular components sum to 1. Among the 1000 proportion matrices (*K* by *J*; *K* refers to the cellular component number, and J refers to the sample number), we picked the one weight matrix that has the smallest condition number.

“Real” refers to the weight matrix that mimics immune cell compositions in the real whole blood sample. We sampled weights from the uniform distribution with min and max values defined by previous observations of blood samples [21]. A small modification on the weights of myeloid DC and CD4 T cells was made to compensate for other missing cell types in the blood and the investigation of multicollinearity issue. The exact modification is available in our source code at https://github.com/LiuzLab/paper_deconvBenchmark (DOI:10.5281/zenodo.4521514). We simulated 1000 weights (*K* by 1000, *K* refers to the cellular component number) where the weights of each cellular component are sampled from the predefined range. In the initial weight matrix, we picked *J* (equals to the sample number) number of columns whose sum is closest to 1 and retrieved a *K* by *J* weight matrix. The above filtering process is added to avoid violation of the predefined range after rescaling. After weight simulation, the range of each component is checked and only weights that satisfied both sum-to-1 and predefined range is selected.

“Dominant” refers to a weight matrix with one major component and *K*-1 minor components. The major component was sampled from the uniform distribution within the range of 0.9–0.99. Minor components were sampled within the range $\frac{1-{p}_{\mathrm{major}}}{K-1}$ to $\frac{1-{p}_{\mathrm{major}}}{K-1}+0.01$. After the initial simulation, all weights are rescaled to meet the sum-to-1 restriction.

“Uniform” refers to a weight matrix with all components have weights at a similar level. All cellular components are sampled within the range $\frac{1}{K}$ to $\frac{1}{K}+0.04$. After the initial simulation, all weights are rescaled to meet the sum-to-1 restriction.

#### Sim3

In this benchmarking framework, we studied the impact of unknown biological content and measurement scales (Table 1 and Additional file 1: Figure S2b). To study unknown biological content, we generated mixtures with tumor spike-ins (HCT 116) [34]. In total, we created three sets of tumor spike-ins: small, large, and mosaic. Tumor proportions are sampled from uniform distributions and only differ in parameters used to set minimum and maximum values in the sampling. “Small” tumor spike-ins are sampled within the range of 0.2–0.3, “large” tumor spike-ins are sampled within the range of 0.7–0.8, and “mosaic” tumor spike-ins are sampled within the range of 0.05–0.95. We then added three sets of tumor spike-in proportions to the weight matrices generated in the Sim2 and rescaled them to have proportions of all components sum to 1. After defining weights, we performed in silico mixing in the count unit and then normalized it to other quantification units (cpm, tpm, and countNorm). To study the impact of the measurement scale, we generated two sets of evaluations where one used absolute proportions of immune components as the ground truth and the other used relative proportions of immune components as the ground truth. The toy example of the absolute measurement scale and the relative measurement scale is in Additional file 2: Table S4. We used GSE60424 [30], GSE64655 [31], GSE51984 [32], GSE115736 [33], and GSE118490 [34] data to generate Sim3.

### Assessment of deconvolution performance

To evaluate the performances of deconvolution methods, we used Pearson correlation coefficient and mean absolute deviance as evaluation metrics. Evaluation metrics of one cell type in a mixture set are calculated based on the following equations:

Pearson correlation coefficient (*r*):

$$ \frac{\sum \limits_{j=1}^J\left({x}_j-\overline{x}\right)\left({y}_j-\overline{y}\right)}{\sqrt{\sum \limits_{j=1}^J{\left({x}_j-\overline{x}\right)}^2\sum \limits_{j=1}^J{\left({y}_j-\overline{y}\right)}^2}} $$

Mean absolute deviance (mAD):

$$ \frac{\sum \limits_{j=1}^J\mid {x}_j-{y}_j\mid }{J} $$

where *j* is the sample index, and *J* is the total number of mixture samples in a dataset. *x*_*j*_ is the estimated cellular proportion of sample *j*, and *y*_*j*_ is the ground truth of sample *j*. When a deconvolution returns NA values, we directly assign the highest penalty for the evaluation metrics: *r* = −1, and mAD = 1, which is the worst possible value. NA values can arise from two sources: deconvolution algorithm and correlation coefficient calculation. For instance, we found that in the Sim1 analysis, CAMfree (reference-free deconvolution method) [14] will return NAs when it fails to converge. To consider this type of failure in the final comparisons, we converted NAs to − 1 or 1, which is the worst correlation outcome (Additional file 1: Figure S3) and the worst mAD outcome (Additional file 1: Figure S4). Correlation calculation can also produce NA values when the denominator of the Pearson’s correlation coefficient equation is zero. This type of error usually occurred in Sim2 and Sim3, in which methods like LinSeed [18] and DeconvRNASeq [16] returned all zero estimations for a single cell type (Additional file 1: Figure S13).

## Supplementary Information

**Additional file 1: Figures S1–S18**, the outline of simulation frameworks, evaluation results based on three benchmarking frameworks, variance analysis based on real and simulated data.

**Additional file 2: Tables S1–S4**, organized description of the benchmarking framework, tested factors, tested methods, and measurement scales.

**Additional file 3:** Data description with the GEO accession number.

**Additional file 4:** Review history.

## Data Availability

All raw sequencing data in .sra format can be downloaded from GEO. The corresponding GEO accession number for each analysis is listed in Table 1. We also put the data description of all raw data source in Additional file 3. All codes are available under the MIT license at the https://github.com/LiuzLab/paper_deconvBenchmark [35] (DOI:10.5281/zenodo.4521514).
