# Supplementary material for: A benchmark for RNA-seq deconvolution analysis under dynamic testing environments
Source: Genome Biol. 2021 Apr 12;22:102. doi: 10.1186/s13059-021-02290-6 (PMC8042713; doi:10.1186/s13059-021-02290-6)
Supplement: Supplementary file 1 — Additional file 1: Figures S1–S18, the outline of simulation frameworks, evaluation results based on three benchmarking frameworks, variance analysis based on real and simulated data. [file 13059_2021_2290_MOESM1_ESM.pdf]

Supplementary Figures

Figure S1 - Outline of benchmarking framework Sim1

Figure S2 - Outline of benchmarking framework Sim2 and Sim3

Figure S3 - Correlation heatmaps of Sim1_simModel

Figure S4 - mAD heatmaps of sim1_simModel

Figure S5 - Evaluation results of sim1_simModel based on mAD

Figure S6 – Variance analysis of GSE60424

Figure S7 – Variance analysis of GSE113590

Figure S8 - Scatter plots of simulated profiles in Sim1_simModel

Figure S9 - Mean-variance plots of simulations in Sim1_simModel

Figure S10 - Density plots of CV values in Sim1_simModel

Figure S11 – Evaluation results of Sim1_libSize based on mAD

Figure S12 - Evaluation results of Sim2 based on mAD

Figure S13 - Cell-type-specific evaluation heatmaps of Sim2 based on correlation

Figure S14 - Cell-type-specific evaluation heatmaps of Sim2 based on mAD

Figure S15 - Scatter plots of estimated weights vs. ground truths of mixtures with 10 cellular components.

Figure S16 - Summarized mAD heatmaps of Sim3

Figure S17 - Summarized evaluation heatmaps of Sim3 in the relative scale

Figures S18 - Summarized evaluation heatmaps of Sim3 in the absolute scale

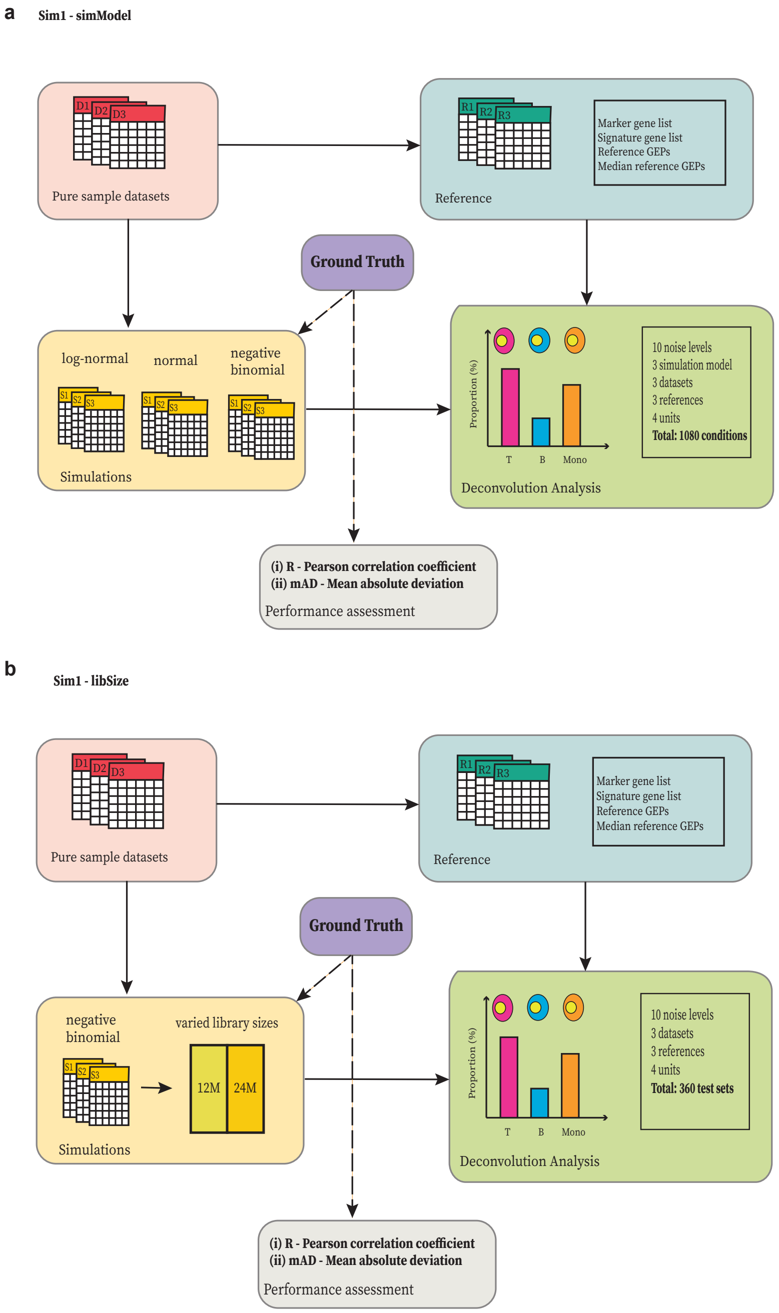

Figure S1| Outline of benchmarking framework Sim1

a, Outline of benchmarking framework Sim1_simModel. **b,** Outline of benchmarking framework Sim1_libSize

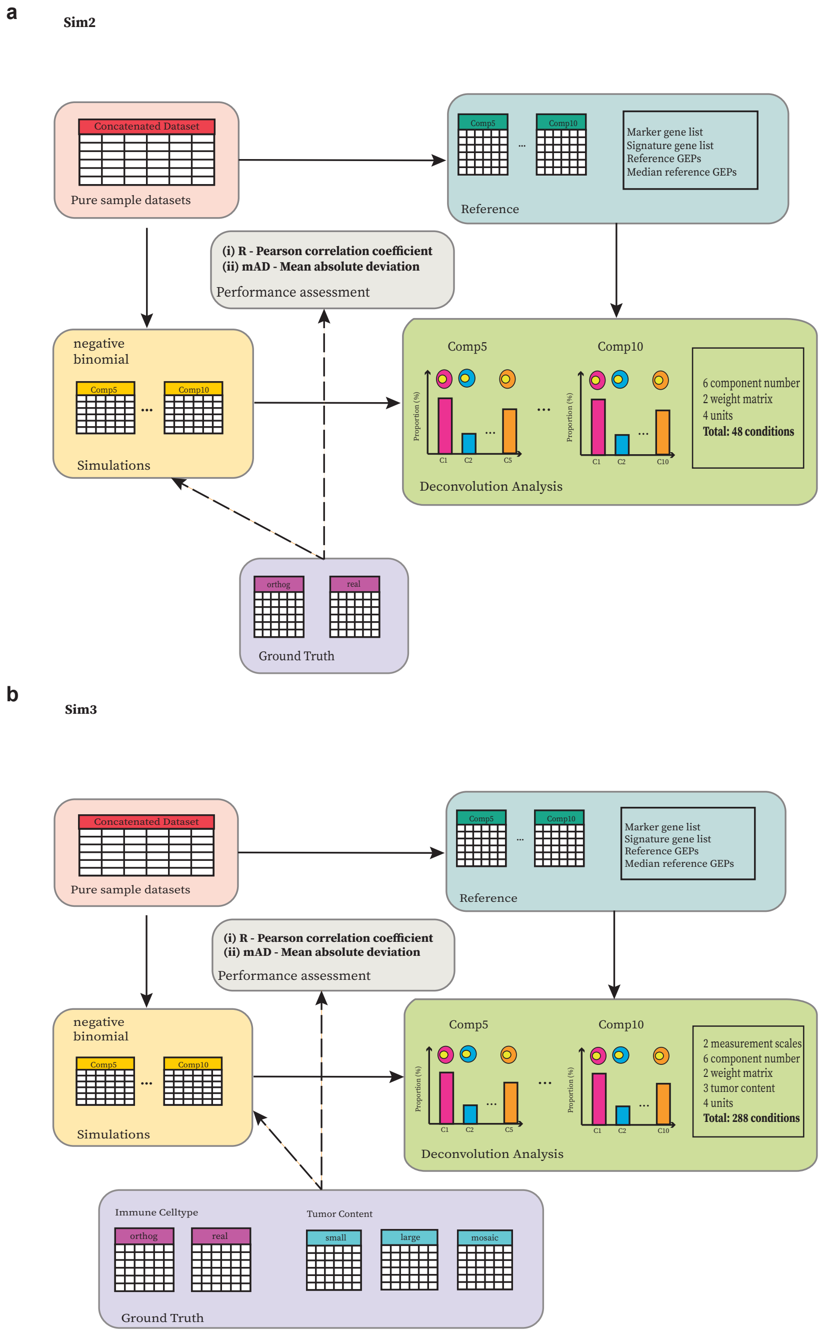

Figure S2| Outline of benchmark frameworks Sim2 and Sim3

a, Outline of benchmarking framework Sim2. **b,** Outline of benchmarking framework Sim3.

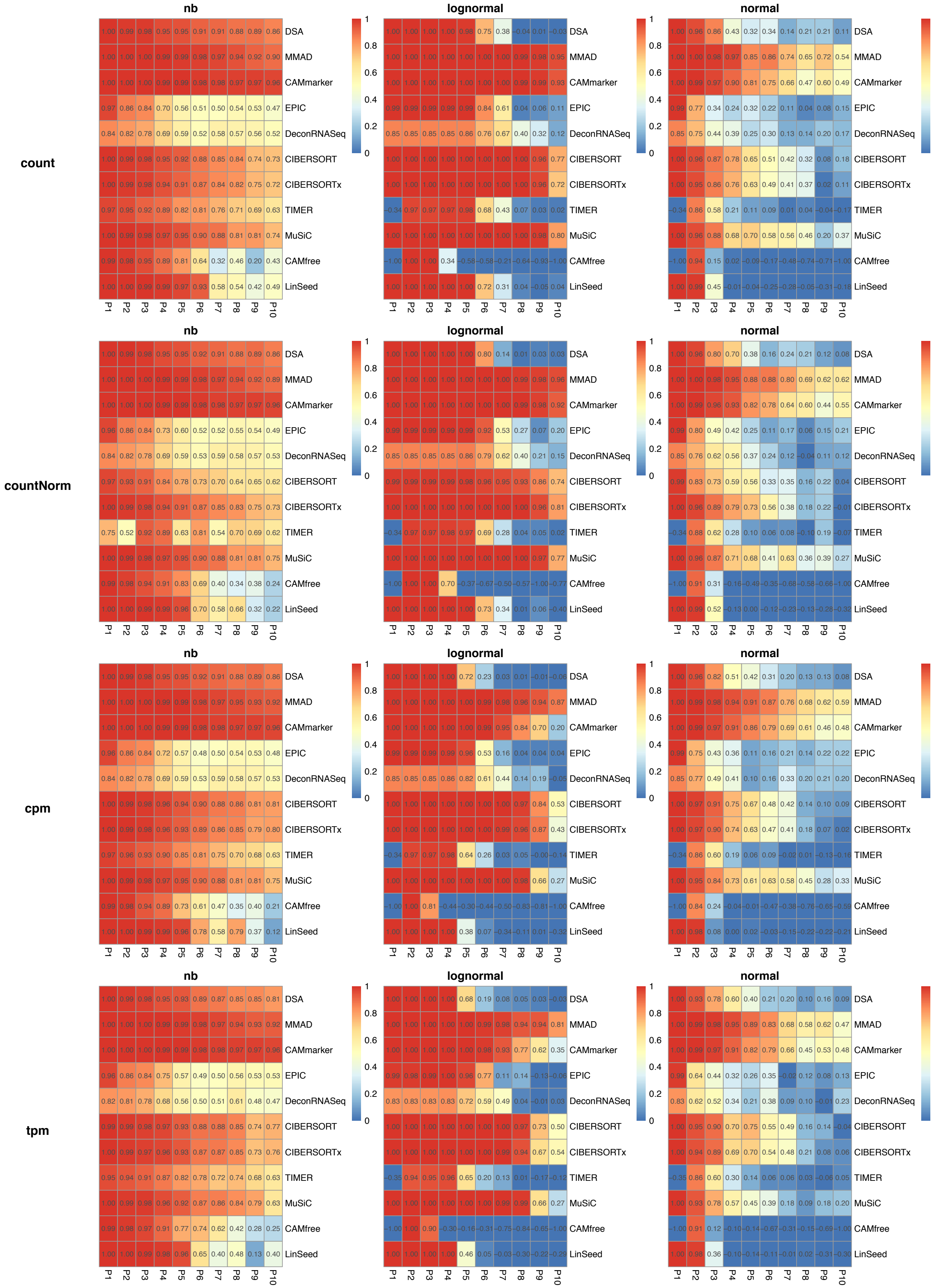
nb
lognormal
normal
count
countNorm
cpm
tpm
DSA
MMAD
CAMmarker
EPIC
DeconRNASeq
CIBERSORT
CIBERSORTx
TIMER
MuSiC
CAMfree
LinSeed
P1
P2
P3
P4
P5
P6
P7
P8
P9
P10
1
0.8
0.6
0.4
0.2
0

Figure S3| Correlation heatmaps of sim1_simModel

The deconvolution results of Sim1_simModel are organized by 12 heatmaps where each row panel refers to the results derived from the same quantification unit and column panel refers to the results derived from the same simulation model. In each heatmap, row indexes refer to the deconvolution methods and column indexes refer to the noise levels. Each cell in the heatmap indicates averaged correlations of 9 testing sets (3 mixture sets $\times$ 3 reference sets)(Methods).

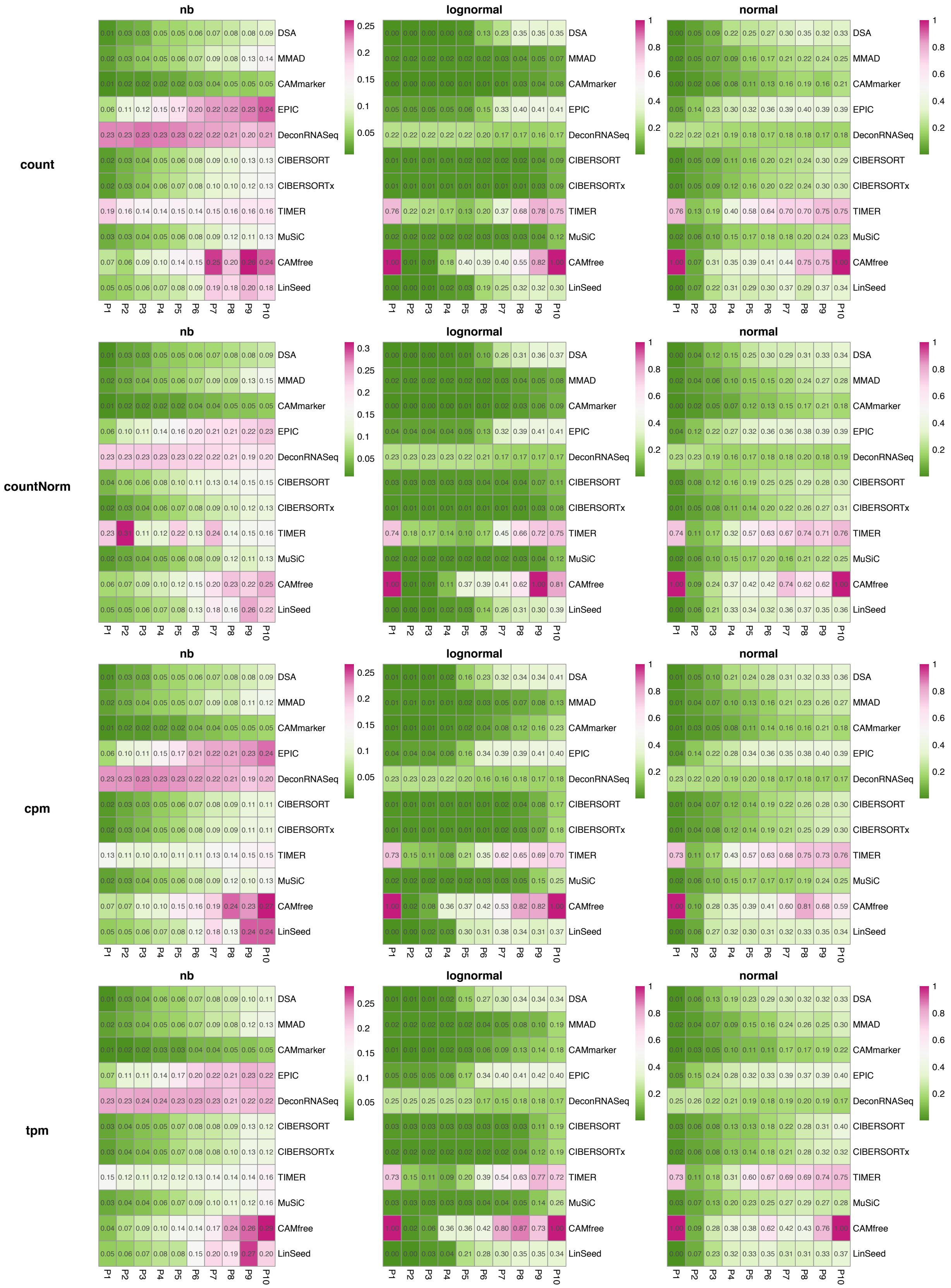
nb
lognormal
normal
count
countNorm
cpm
tpm
DSA
MMAD
CAMmarker
EPIC
DeconRNASeq
CIBERSORT
CIBERSORTx
TIMER
MuSiC
CAMfree
LinSeed
P1
P2
P3
P4
P5
P6
P7
P8
P9
P10
0.25
0.2
0.15
0.1
0.05
0.3
1
0.8
0.6
0.4
0.2

Figure S4| mAD heatmaps of sim1_simModel

The deconvolution results of Sim1_simModel are organized by 12 heatmaps where each row panel refers to the results derived from the same quantification unit and each column panel refers to the results derived from the same simulation model. In each heatmap, row indexes refer to the tested deconvolution methods and column indexes refer to the noise levels. Each cell in the heatmap indicates averaged mADs of 9 testing sets (3 mixture sets × 3 reference sets)(Methods).

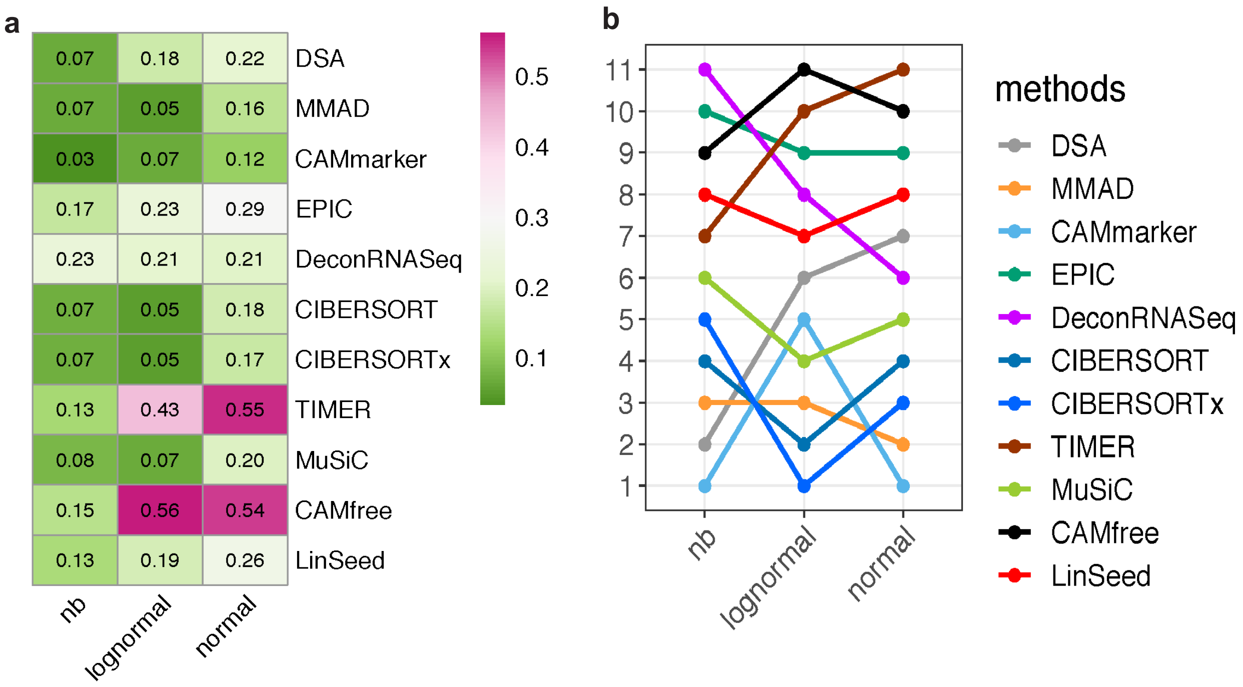

Figure S5| Evaluation results of sim1_simModel based on mAD

a, Heatmap of summarized evaluation results based on the mADs and **b,** rankings of tested deconvolution methods. In each heatmap, row indexes refer to the tested methods and column indexes refer to the simulation models (negative binomial, log-normal, and normal).

Variance Structure of GSE60424

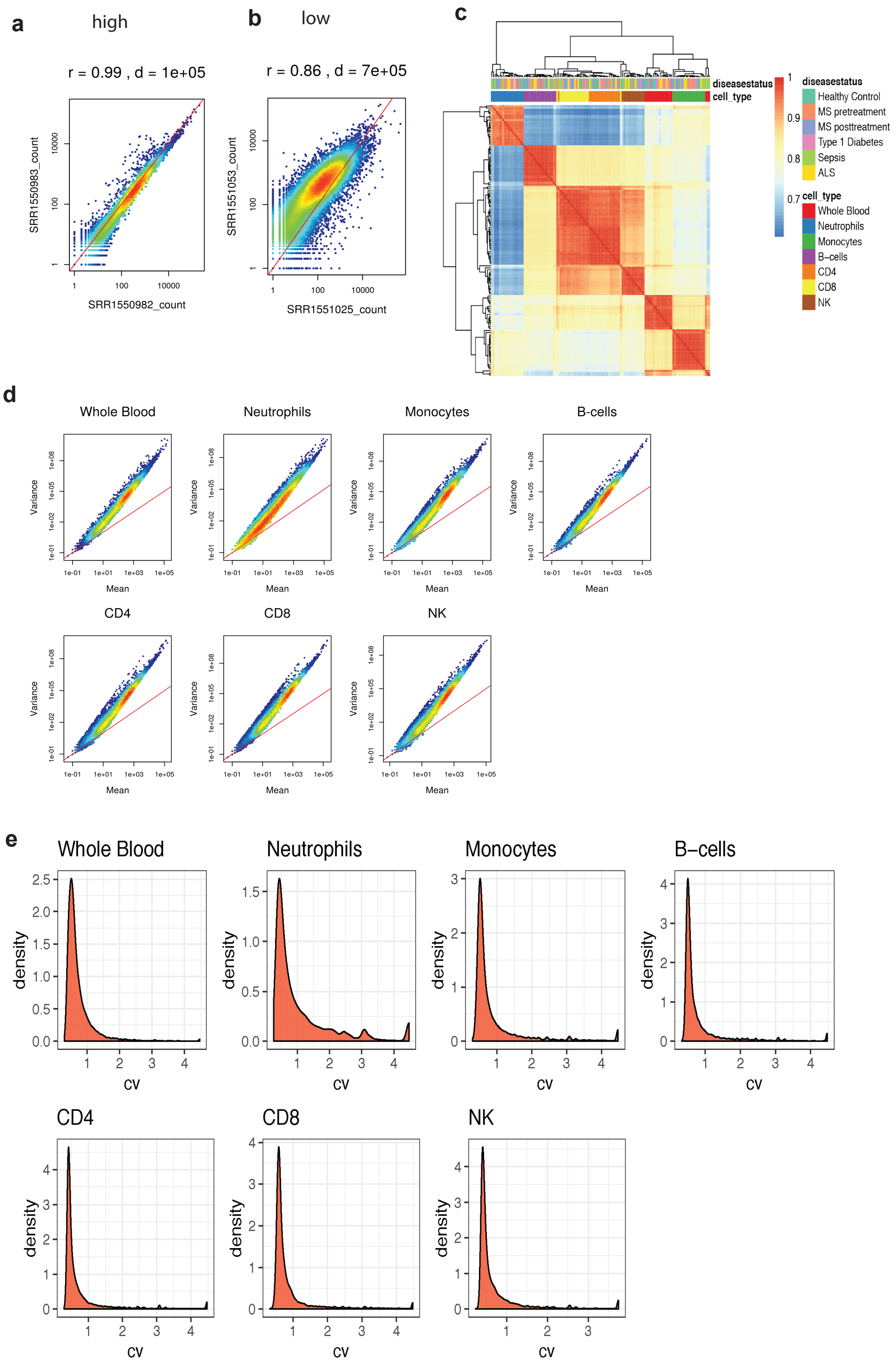

Figure S6| Variance analysis of GSE60424

a,b, Scatter plots of whole blood sample profiles with (**a**) highest correlation and (**b**) lowest correlation. **c,** Correlation heatmap across all sample pairs. **d,** Mean-variance plot **e,** Density plot of CV (coefficient of variance) r: Spearman correlation coefficient, d: Euclidian distance. All points are plotted in the log space. (All results are in the count unit.)

Variance Structure of GSE113590

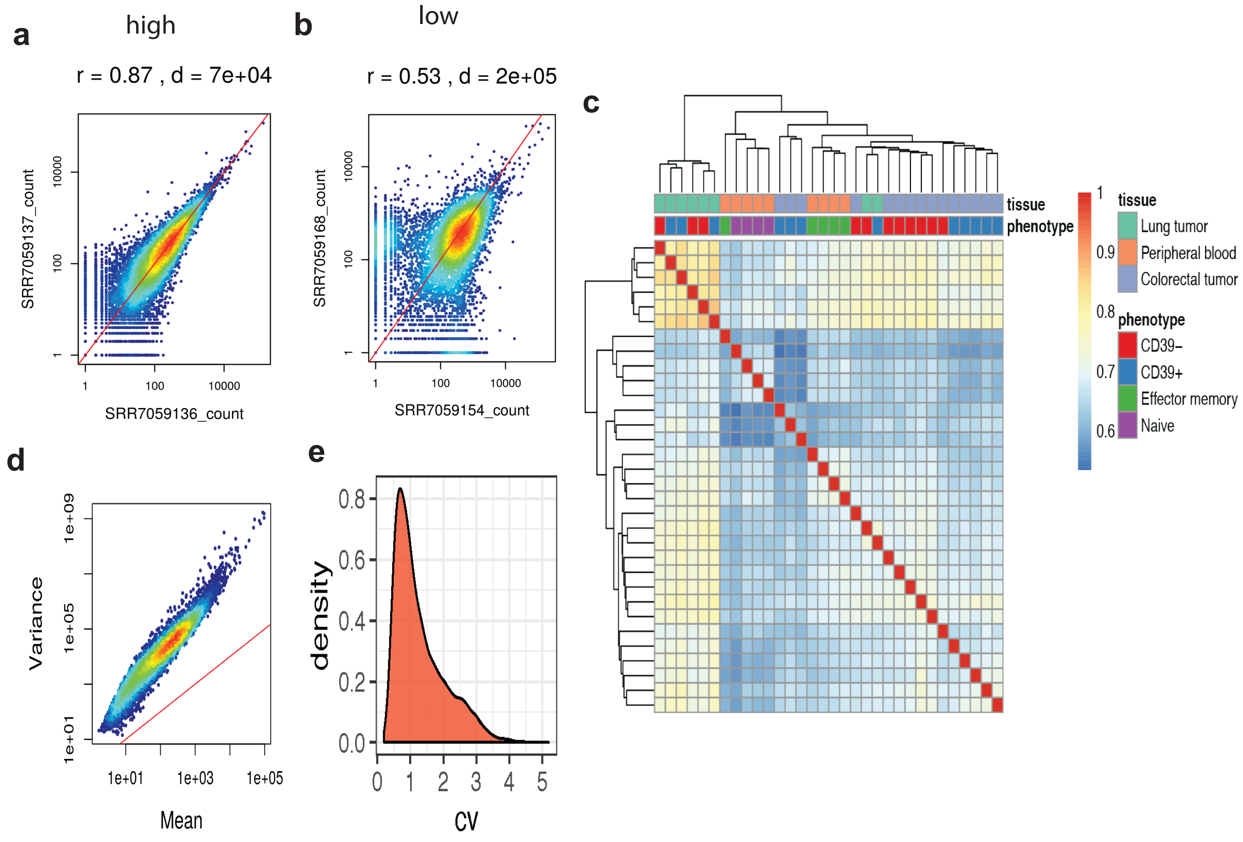

Figure S7| Variance analysis of GSE113590

a,b, Scatter plots of CD8 T sample profiles with (**a**) highest correlation and (**b**) lowest correlation. **c,** Correlation heatmap across all sample pairs. **d,** Mean-variance plot **e,** Density plot of CV (Coefficient of variance) r: Spearman correlation coefficient, d: Euclidian distance. All points are plotted in the log space. (All results are in the count unit.)

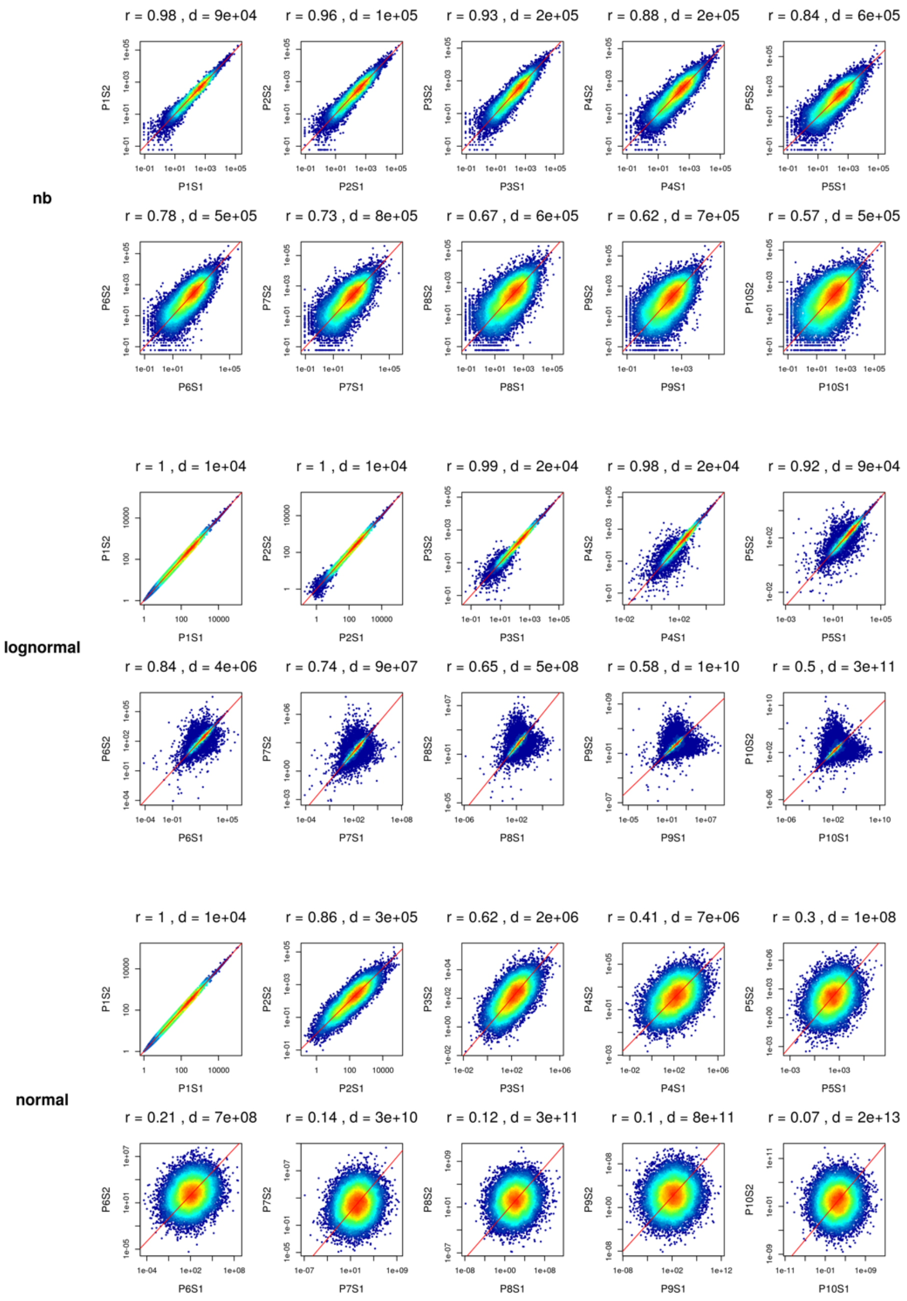

Figure S8| Scatter plots of simulated profiles in Sim1_simModel

Sample-sample scatter plots of the first two simulated profiles that are derived from GSE51984. Each row panel refers to the simulation model used to generate simulation. (All results are in the count unit.)

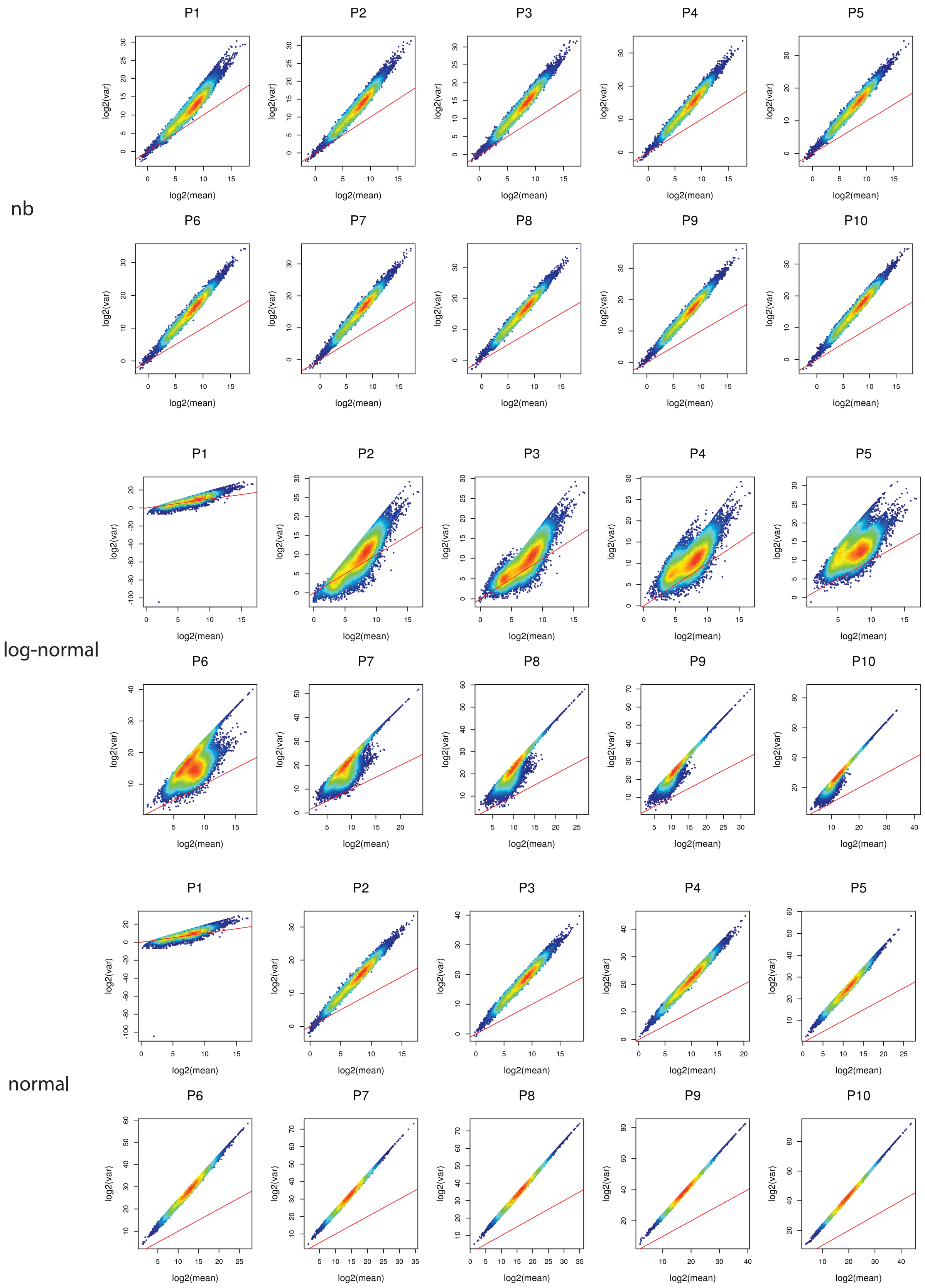
nb
log-normal
normal
P1
P2
P3
P4
P5
P6
P7
P8
P9
P10
log2(var)
log2(mean)

Figure S9| Mean-variance plots of simulations in Sim1_simModel

Mean-variance plots of simulations derived from negative binomial (nb), log-normal, and normal simulation models. In each simulation model, 10 plots are generated for 10 noise levels (P1–P10). The simulations in this figure are generated from GSE51984. (All results are in the count unit.)

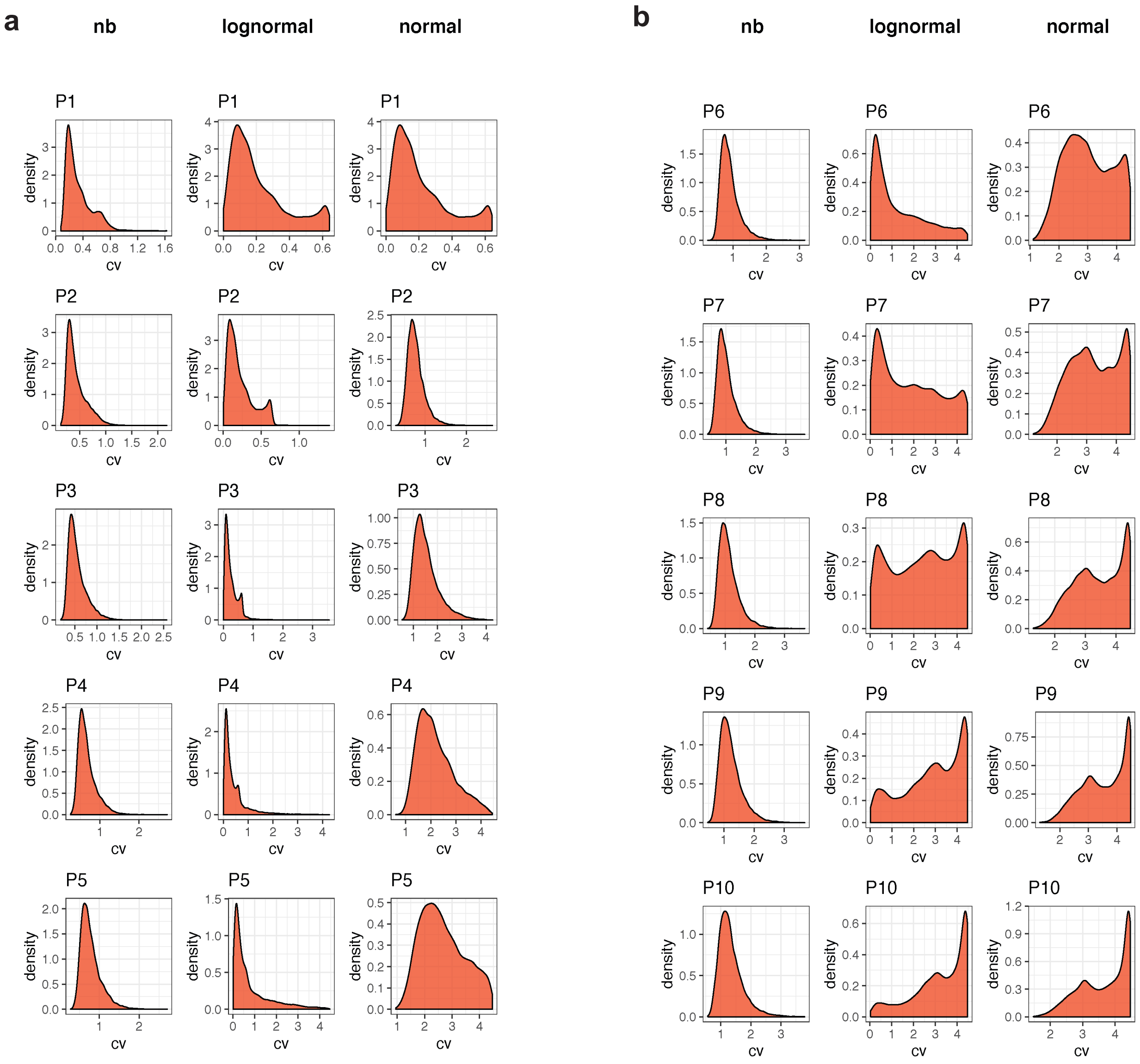

Figure S10| Density plots of CV values in Sim1_simModel

a, b, Density curve of CV (Coefficient of variance) from 3 simulation models with noise level (**a**) P1 – P5 and (**b**), P6 – P10. The simulations in this figure are generated from GSE51984. (All results are in the count unit.)

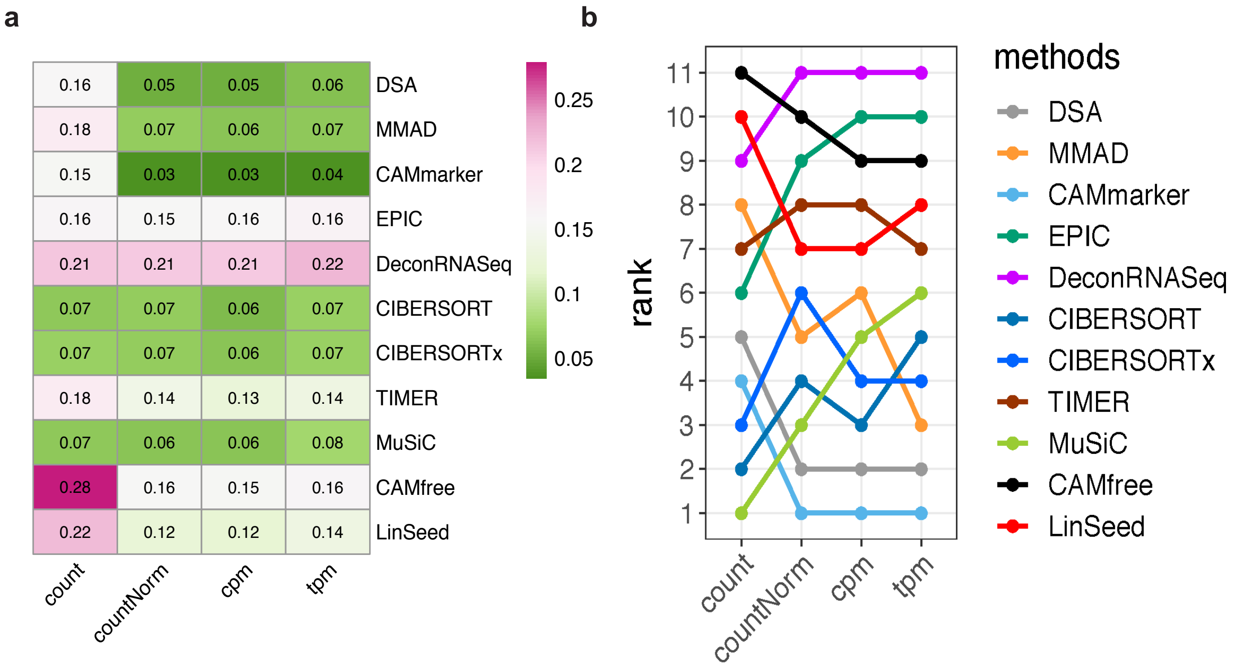

Figure S11| Evaluation results of Sim1_libSize based on mAD

a, Heatmap of summarized evaluation results based on the mADs and **b,** rankings of tested deconvolution methods. In each heatmap, row indexes refer to the tested methods and column indexes refer to the quantification units (count, countNorm, cpm, and tpm).

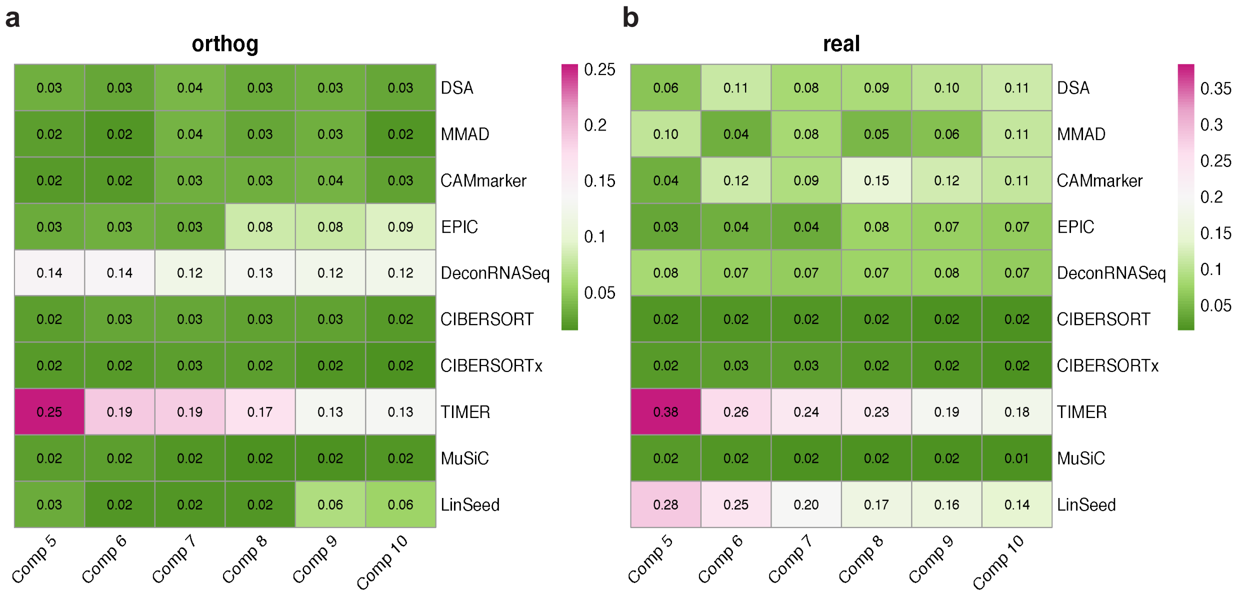

Figure S12| Evaluation results of Sim2 based on mAD

a,b, Heatmaps of summarized evaluation results based on the mAD metric with (**a**) 'orthog' weight matrix and (**b**) 'real' weight matrix. In each heatmap, row indexes refer to the tested methods and column indexes refer to the cellular component numbers.

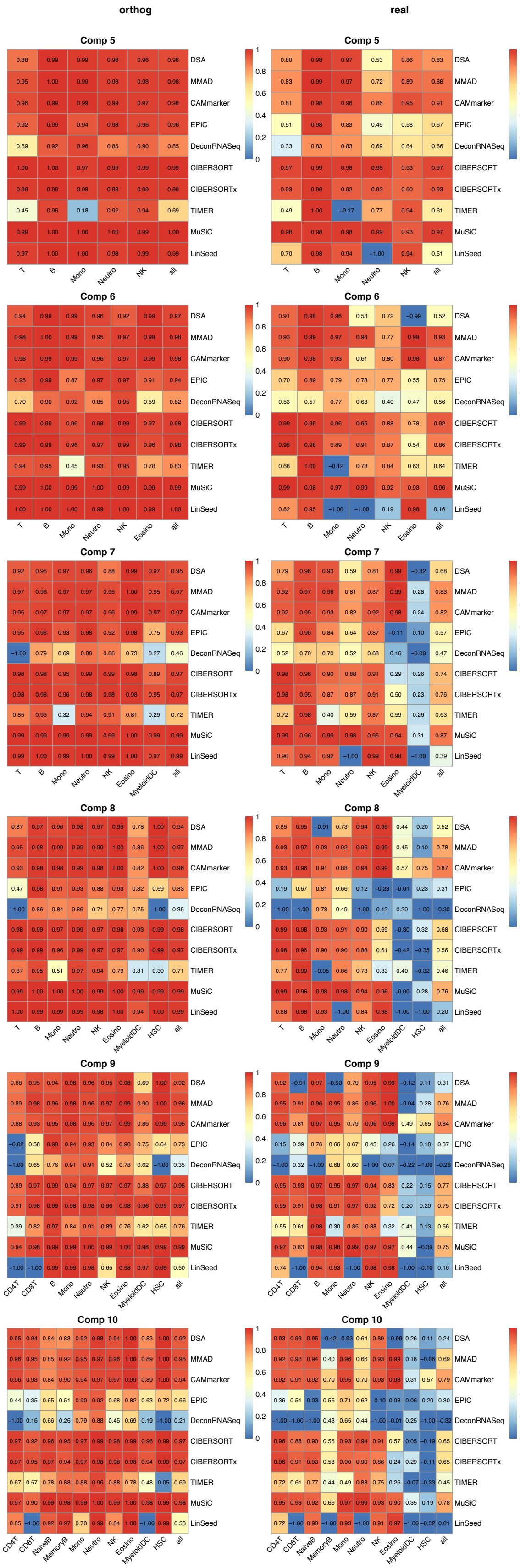
orthog
real
Comp 5
Comp 6
Comp 7
Comp 8
Comp 9
Comp 10
DSA
MMAD
CAMmarker
EPIC
DeconRNASeq
CIBERSORT
CIBERSORTx
TIMER
MuSiC
LinSeed
T
CD4T
CD8T
B
NaiveB
MemoryB
Mono
Neutro
NK
Eosino
MyeloidDC
HSC
all

Figure S13| Cell-type-specific evaluation heatmaps of Sim2 based on correlation

Evaluation results of Sim2 based on the correlation metric. Each row panel indicates the number of cellular components in the mixture and each column panel indicates the weight matrix type ('orthog' vs. 'real'). In each heatmap, row indexes refer to the tested methods, column indexes refer to the cell types, and the last column 'all' refers to the averaged evaluation results across all cell types.

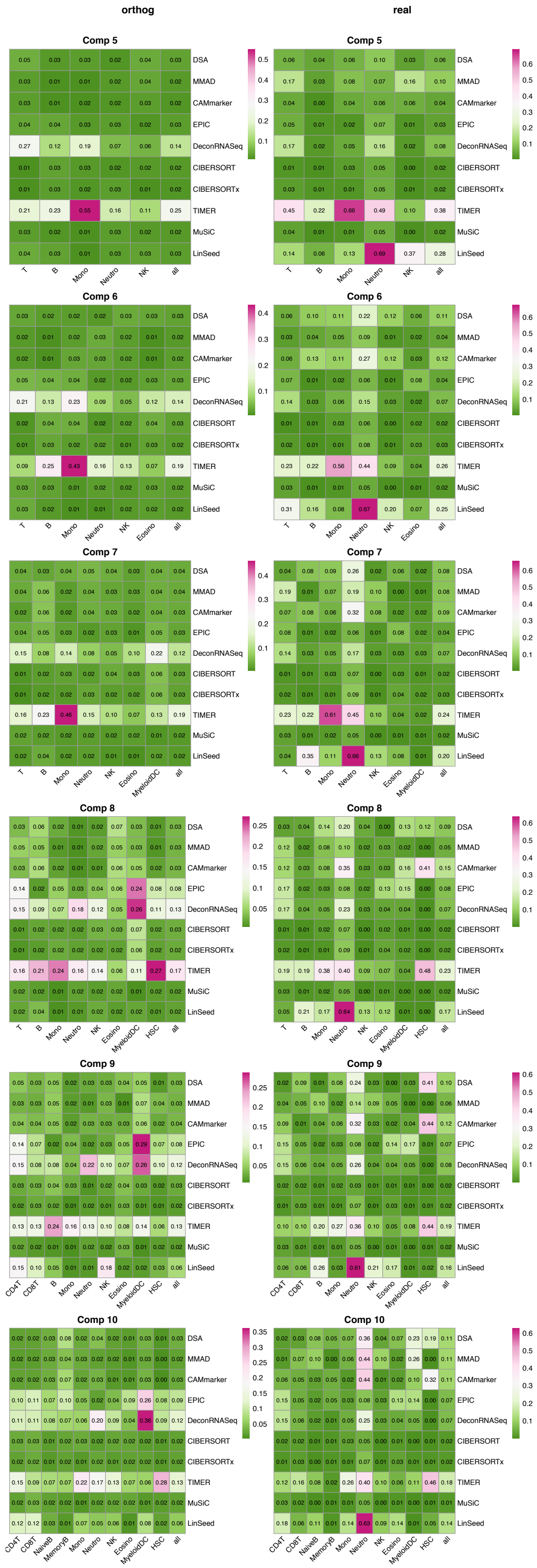
orthog
real
Comp 5
Comp 6
Comp 7
Comp 8
Comp 9
Comp 10
DSA
MMAD
CAMmarker
EPIC
DeconRNASeq
CIBERSORT
CIBERSORTx
TIMER
MuSiC
LinSeed
T
CD4T
CD8T
NaiveB
MemoryB
B
Mono
Neutro
NK
Eosino
MyeloidDC
HSC
all

Figure S14| Cell-type-specific evaluation heatmaps of Sim2 based on mAD

Evaluation results of Sim2 based on the mAD metric. Each row panel indicates the number of cellular components in the mixture and each column panel indicates the weight matrix type (‘orthog’ vs. ‘real’). In each heatmap, row indexes refer to the tested methods, column indexes refer to the cell types, and the last column ‘all’ refers to the averaged evaluation results across all cell types.

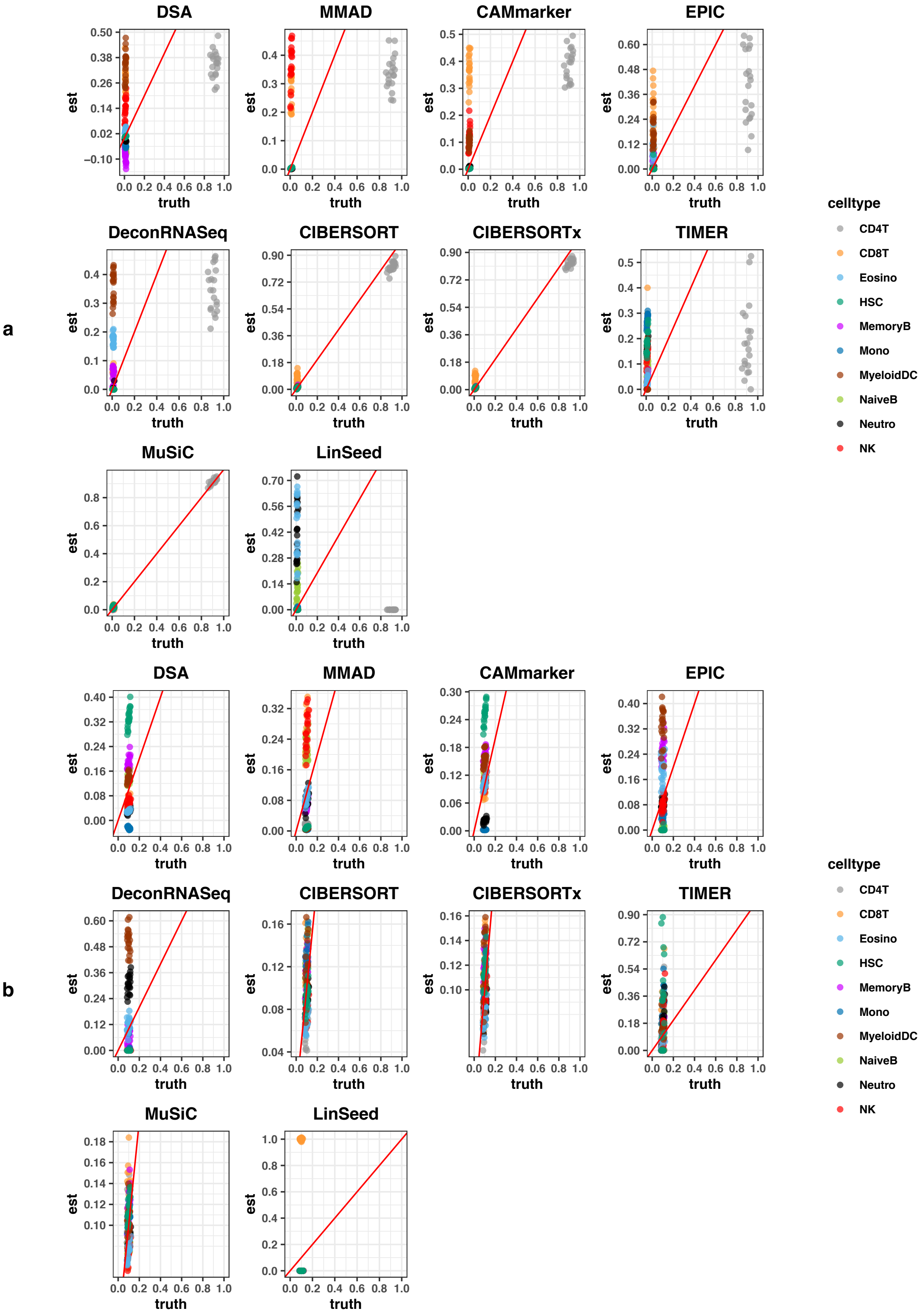
a
DSA
MMAD
CAMmarker
EPIC
DeconRNASeq
CIBERSORT
CIBERSORTx
TIMER
MuSiC
LinSeed
est
truth
celltype
CD4T
CD8T
Eosino
HSC
MemoryB
Mono
MyeloidDC
NaiveB
Neutro
NK
b
DSA
MMAD
CAMmarker
EPIC
DeconRNASeq
CIBERSORT
CIBERSORTx
TIMER
MuSiC
LinSeed
est
truth
celltype
CD4T
CD8T
Eosino
HSC
MemoryB
Mono
MyeloidDC
NaiveB
Neutro
NK

Figure S15| Scatter plots of estimated weights vs. ground truths of mixtures with 10 cellular components. a, Mixture sets with 'dominant' weight matrix . b, Mixture sets with 'uniform' weight matrix. For more detail of weight matrix construction, please refer to the Method section.

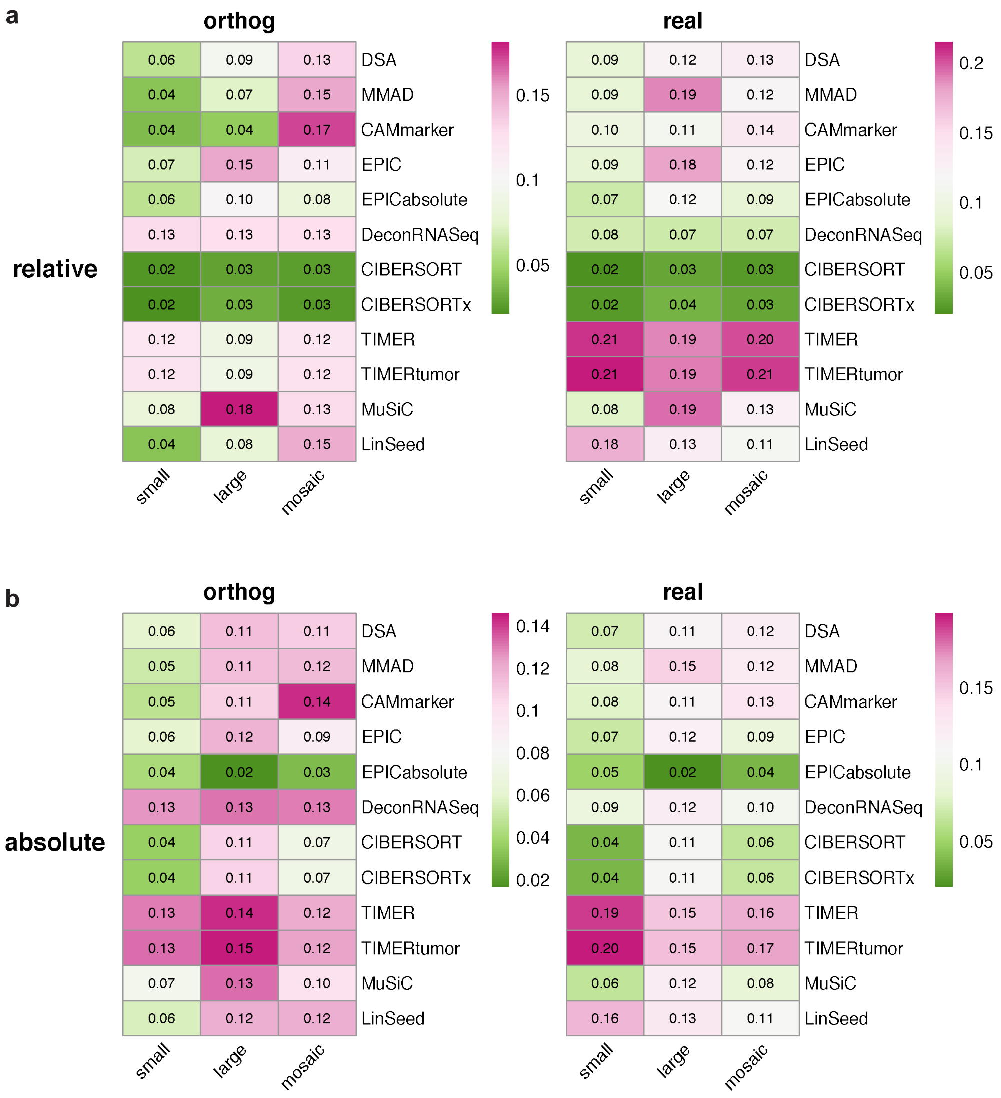

Figure S16| Summarized mAD heatmaps of Sim3

a,b, Heatmaps of summarized evaluation metric based on mAD evaluation metric with (**a**) relative measurement scale and (**b**) absolute measurement scale. In each heatmap, row indexes refer to the tested methods, and column indexes refer to the types of tumor spike-ins (small, large, and mosaic).

a

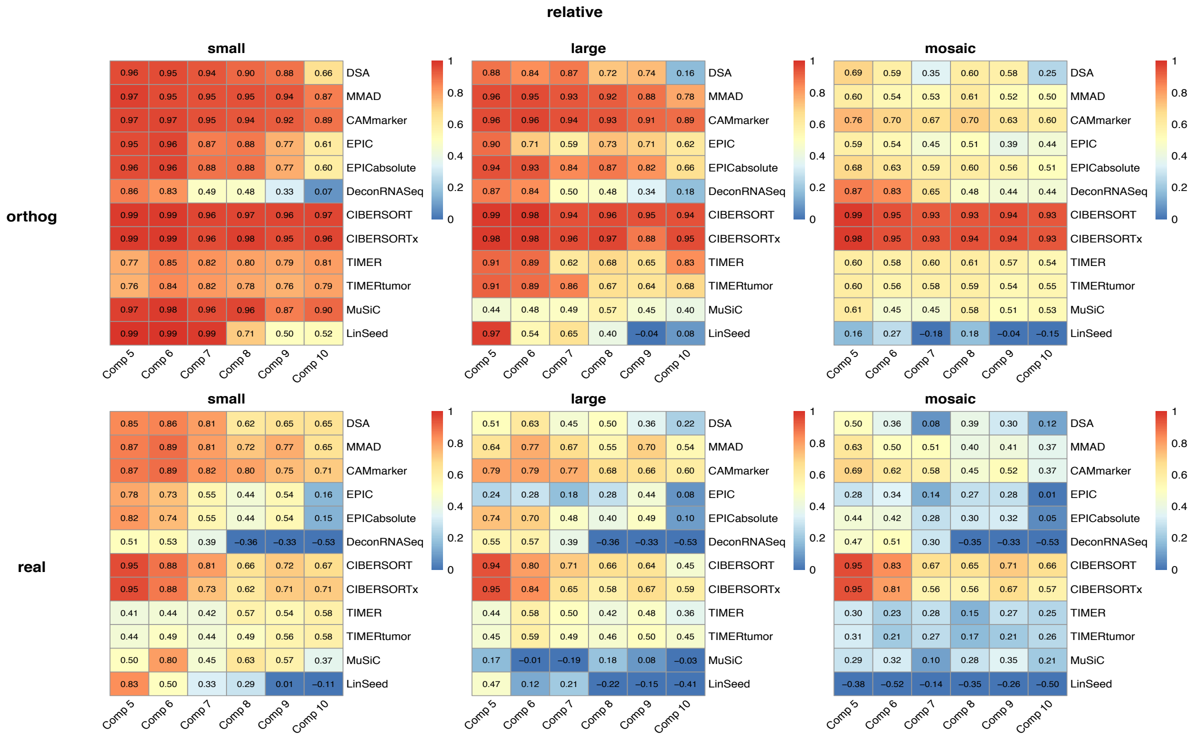
relative
orthog
real
small
large
mosaic
DSA
MMAD
CAMmarker
EPIC
EPICabsolute
DeconRNASeq
CIBERSORT
CIBERSORTx
TIMER
TIMERtumor
MuSiC
LinSeed
Comp 5
Comp 6
Comp 7
Comp 8
Comp 9
Comp 10

b

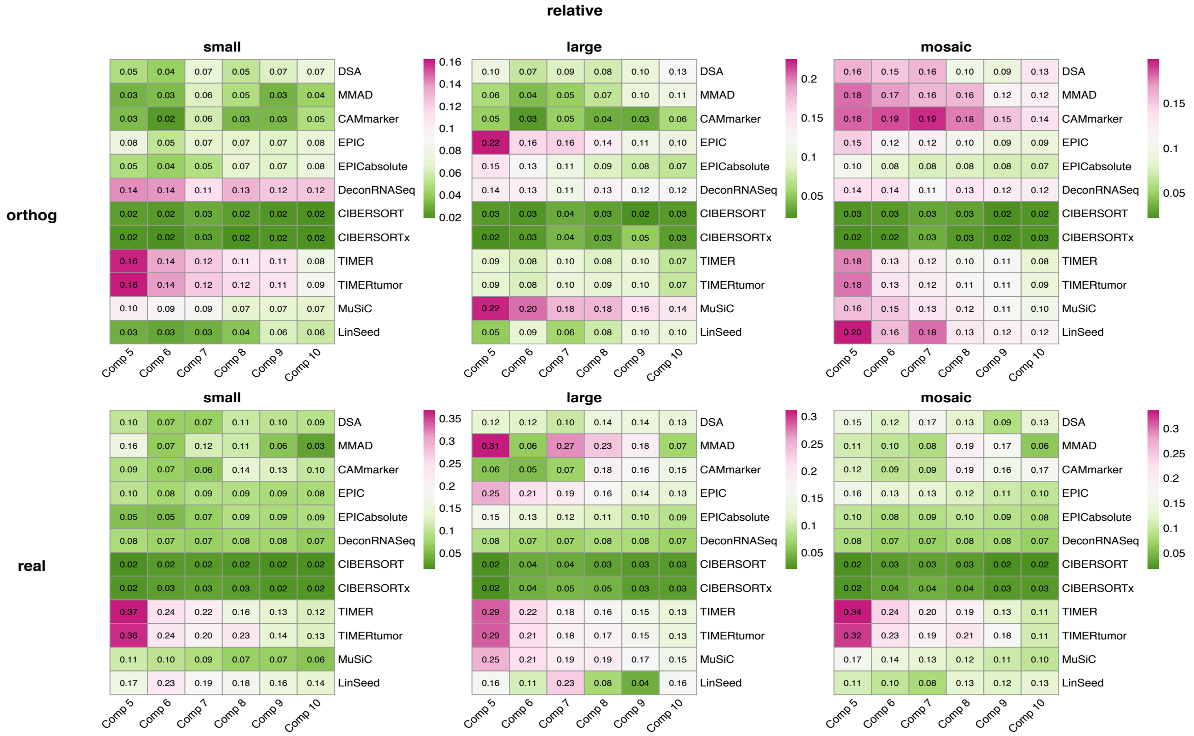
relative
orthog
real
small
large
mosaic
DSA
MMAD
CAMmarker
EPIC
EPICabsolute
DeconRNASeq
CIBERSORT
CIBERSORTx
TIMER
TIMERtumor
MuSiC
LinSeed
Comp 5
Comp 6
Comp 7
Comp 8
Comp 9
Comp 10

Figure S17| Summarized evaluation heatmaps of Sim3 in the relative scale

a, b, Summarized evaluation heatmap based on (**a**) Pearson's correlation coefficients and (**b**) mAD. The row panel indicates the type of weight matrix and the column panel indicates the type of tumor spike-ins (small, large, and mosaic). In each heatmap, row indexes refer to the tested methods and column indexes refer to the number of cellular components in the mixture.

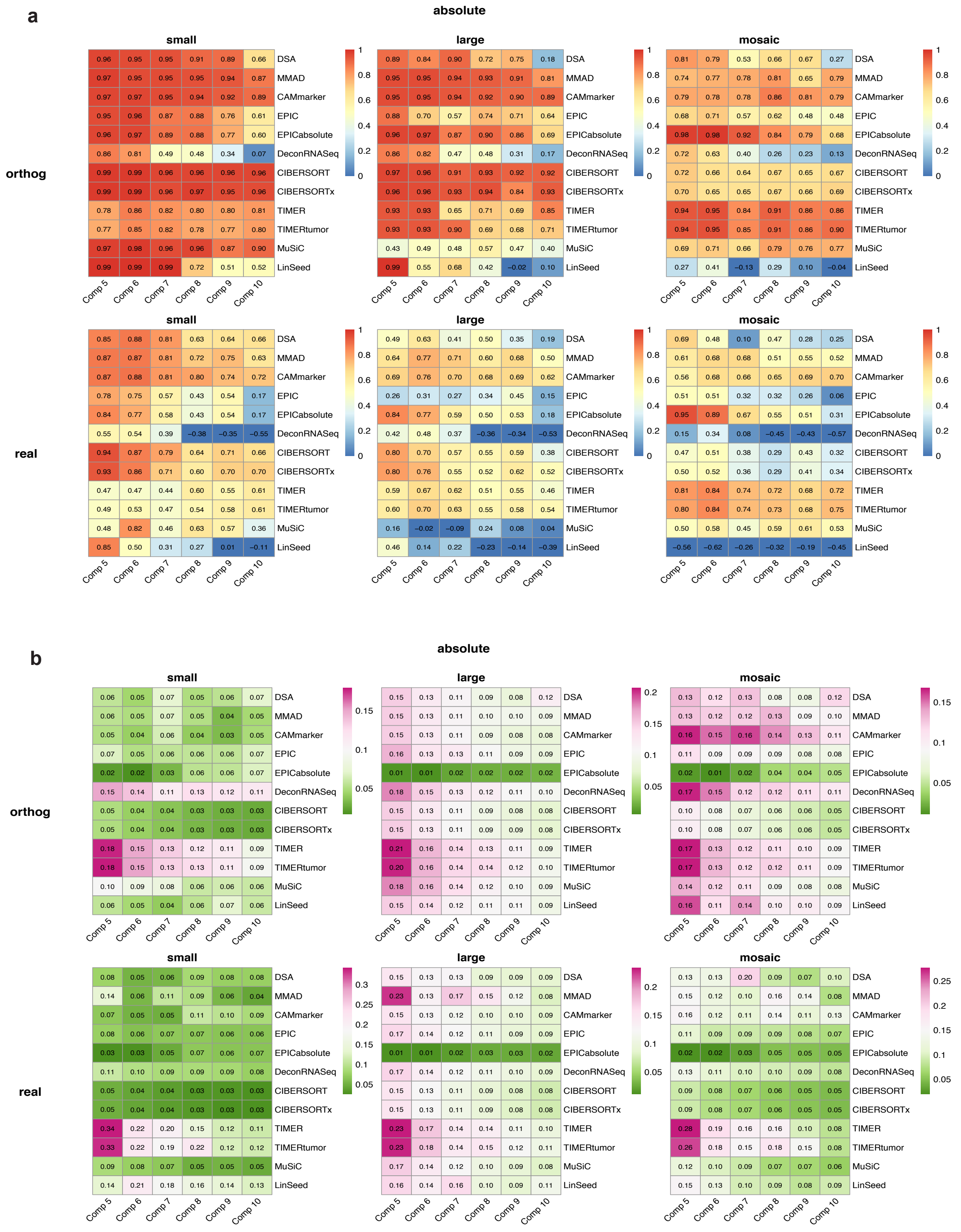
a
absolute
orthog
real
small
large
mosaic
DSA
MMAD
CAMmarker
EPIC
EPICabsolute
DeconRNASeq
CIBERSORT
CIBERSORTx
TIMER
TIMERtumor
MuSiC
LinSeed
Comp 5
Comp 6
Comp 7
Comp 8
Comp 9
Comp 10
1
0.8
0.6
0.4
0.2
0
b
absolute
orthog
real
small
large
mosaic
0.15
0.1
0.05
0.2
0.3
0.25

Figure S18| Summarized evaluation heatmaps of Sim3 in the absolute scale

a, b, Summarized evaluation heatmap based on (**a**) Pearson's correlation coefficients and (**b**) mAD. The row panel indicates the type of weight matrix and the column panel indicates tumor spike-ins (small, large, and mosaic). In each heatmap, row indexes refer to the tested methods and column indexes refer to the number of cellular components in the mixture.
