# Supplementary material for: A benchmark for RNA-seq deconvolution analysis under dynamic testing environments
Source: Genome Biol. 2021 Apr 12;22:102. doi: 10.1186/s13059-021-02290-6 (PMC8042713; doi:10.1186/s13059-021-02290-6)
Supplement: Supplementary file 2 — Additional file 2: Tables S1–S4, organized description of the benchmarking framework, tested factors, tested methods, and measurement scales. [file 13059_2021_2290_MOESM2_ESM.pdf]

Supplementary Tables

Table S1 - Three benchmarking frameworks commonly applied

Table S2 – Technical and biological factors studied in each benchmarking framework

Table S3 – Deconvolution methods tested in this study

Table S4 – A toy example of absolute and relative measurement scales

Table S1: Three benchmarking frameworks commonly applied

**Framework type**	**Sample source**	**Ground truth**	**Flexibility**	**Cost-effectiveness**	**Similarity to the biological condition**
in silico	simulation	yes	High	High	Low
in vitro	experimentally mixed samples	yes	Medium	Medium	Medium
in vivo	biological specimen	Usually no	Low	Low	High

Table S2: Factors studied in three benchmarking frameworks

Benchmarking frameworks	Factor 1	Factor 2	Factor 3	Factor 4	Factor 5	Number of conditions	Dataset index
**Sim1_simModel**	Simulation model	Noise level	Other noise sources	Units		1080	1,3 and 4
**Sim1_libSize**	Units	Noise level	Other noise sources			360	1,3 and 4
**Sim2**	Weight matrix	Component number	Units			48	1,3,4 and 5
**Sim3**	Tumor content	Measurement scale	Weight matrix	Component number	Units	288	1,3,4,5 and 6
Total number of conditions: 1, 776<br><br>**Summary of factors:**<br>Simulation model: 3 (normal, log-normal, and nb)<br>Noise level: 10 (P1-P10)<br>Units: 4 (count, countNorm, cpm, and tpm)<br>Weight matrix: 2 (orthog and real)<br>Component number: 6 (5 - 10)<br>Tumor content: 3 (small, large, and mosaic)<br>Measurement scale: 2 (relative and absolute)<br>Other noise sources: 9 (variance between reference and mixture)							Data index corresponds to the index in the dataset description

Table S3: Methods tested in this study

Deconvolution methods	Description	Type
DSA	Least squares or quadratic programming	Marker-based
MMAD	Maximum likelihood	Marker-based
CAMmarker	Simplex approach	Marker-based
CIBERSORT	Support vector regression	Reference-based
CIBERSORTx	Support vector regression	Reference-based
EPIC	Weighted least squares	Reference-based
TIMER	Least-squares	Reference-based
DeconRNASeq	Least-squares	Reference-based
MuSiC	Iterative weighted least squares	Reference-based
LinSeed	Simplex approach	Reference-free
CAMfree	Simplex approach	Reference-free

Additional information:

We had to make some modifications to the name of some methods as we used different parameter settings for the same method.

1. CAMmarker and CAMfree are all derived from R package CAMTHC. We named the marker-based approach CAMmarker and the reference-free approach CAMfree.
2. We referred EPIC with the unknown content estimation as EPICabsolute.
3. We referred TIMER with the additional filtering process specific for tumor genes as TIMERtumor.

Table S4: A toy example of absolute and relative measurement scales

	T cell	B cell	Unknown contents	Sum
Absolute scale weights	0.3	0.2	0.5	1
Relative scale weights	0.3/(0.5) = 0.6	0.2/(0.5) = 0.4	/	1
