# Supplementary material for: A benchmark for RNA-seq deconvolution analysis under dynamic testing environments
Source: Genome Biol. 2021 Apr 12;22:102. doi: 10.1186/s13059-021-02290-6 (PMC8042713; doi:10.1186/s13059-021-02290-6)
Supplement: Supplementary file 3 — Additional file 3: Data description with the GEO accession number. [file 13059_2021_2290_MOESM3_ESM.pdf]

Datasets description:

1. **GSE60424 -** Consists of 134 RNA-seq profiles of 6 immune cell types and whole blood from both healthy donors and donors with five immune-associated diseases.
2. **GSE113590 –** Consists of 32 CD8 T cell RNA-seq profiles from peripheral blood, colorectal tumor samples, and lung tumor samples.
3. **GSE64655 -** Consists of 56 RNA-seq profiles of 6 immune cell types and peripheral blood from two vaccinated donors.
4. **GSE51984 –** Consists of 24 RNA-seq profiles of 5 immune cell types and total white blood cells from healthy donors
5. **GSE115736 –** Consists of 42 RNA-seq profiles of 12 immune cell types from healthy donors.
6. **GSE118490 –** HCT116 profiles (unknown tumor content in Sim3)
