# Supplementary material for: A benchmark for RNA-seq deconvolution analysis under dynamic testing environments
Source: Genome Biol. 2021 Apr 12;22:102. doi: 10.1186/s13059-021-02290-6 (PMC8042713; doi:10.1186/s13059-021-02290-6)
Supplement: Supplementary file 4 — Additional file 4: Review history. [file 13059_2021_2290_MOESM4_ESM.docx]

Review History

**First round of review**

**Reviewer 1**

**Are you able to assess all statistics in the manuscript, including the appropriateness of statistical tests used?**

Yes, and I have assessed the statistics in my report.

**Comments to author:**

The present manuscript describes an in silico framework to benchmark computational methods predicting cell type composition from bulk gene expression measurements. The work explores different in silico models to build the artificial mixtures (different models of noise, different models of weight for each cell type, addition of unknown cell types in the mixture) and tests the performance of different tools.

Major comments.

- All the work is restricted to "in silico" generated mixtures of cell type. While this is convenient since the ground truth is directly know, in silico generated mixtures do not reflect the complexity of biological systems (e.g., the fact that reference gene expression profiles change in an often unknown way when mixed together, the use of biologically unrealistic proportions of cell types, the impact of cell type isolation, the variability between different individuals, the impact of different experimental sequencing procedures, the presence of other unknown cell types than only cancer cells, etc., etc.) . As a result, the best methods identified in this work may just be the ones whose underlying model is closest to the model used to computationally generate the mixtures. This is a major limitation of the work, which prevents reaching solid conclusion about the accuracy of a method in a biologically relevant context.

- The authors claim that benchmarking studies are lacking. However, this is not true. First several studies where new methods were developed spent time to find a good framework to robustly validate the results in a biologically relevant context (i.e., not only with artificial mixtures but for instance using single-cell RNA-Seq data, or samples analyzed by flow cytometry or by immuno-histochemistry in parallel to gene expression). Second, several benchmarking studies have been recently published (e.g., Sturm et al Bioinformatics 2019), and many of them are just not cited.

- Throughout the manuscript, it is very difficult to know what kind of cell types are used to build the mixtures, or for other analyses. One example (but this is not the only one) is line 106-109, where there is no indication about what the 'real' data are, and why this analysis enables to discriminate different noise models. Another example is the addition of tumor spike-ins, where I could not find what type of tumor samples have been used (are the authors even sure that these were profiles from pure cancer cells, without cells from the micro-environment?). These issues highlight that the work is mainly computational with some relevant algorithmic/methodological observations, but often quite disconnected from the real issues with biological samples.

- Some cell types are easy to 'deconvolve' (e.g., major immune cell types), while others are much more difficult. This is a major issue in cell type deconvolution, which is almost totally overlooked in the present manuscript.

- Many observations (lower performance with increased noise, with 'real' compared to 'ortholog' weight matrices, with increased number of components) are quite expected and well known in the field and in previous studies.

Other comments:

- CIBSERSORTx should be included in the benchmark.

- The authors should think whether the word 'deconvolution' is really the best. Strictly speaking, deconvolution means predicting not only the fraction of the different cell types, but also the actual gene expression profile for each cell type (something much mode difficult). I'm aware that deconvolution is often used for predicting cell type composition, but maybe worth clarifying this point.

- Line 305: this is actually not best practice in deconvolution analysis, but in building in silico benchmarks for testing deconvolution tools. Quite a different problem...

- It is not always clear how different tools were used. For instance, several tools cannot distinguish memory B cells from naive B cells, but results are still reported for these cell types in Figure 4 for all the tools.

**Reviewer 2**

**Are you able to assess all statistics in the manuscript, including the appropriateness of statistical tests used?**

There are no statistics in the manuscript.

**Comments to author:**

Jin and Liu evaluated the impact of several relevant technical and biological factors in the deconvolution of RNA-seq data by means of constructing three different benchmarking frameworks. The manuscript reads nicely and shows relevant results that researchers working on deconvolution should be aware of.

However, in its current form, I have significant concerns which I think should be taken into consideration and commented in the manuscript:

Primary comments:

-	It is not clear, however, whether they are aware of two highly related studies that have recently appeared: "Comprehensive evaluation of transcriptome-based cell-type quantification methods for immuno-oncology" and "Comprehensive benchmarking of computational deconvolution of transcriptomics data", which cover topics quite similar to the present work. Therefore, I recommend that the authors revise their text to comprehensively relate their discussion to the prior work by others to more clearly indicate what new insights arise from their study.

-	With the exception of "Sim1_simModel", I find the number of mixtures evaluated in the different simulations to be quite limited to produce robust conclusions (e.g. "Tumor spike-ins were introduced to 12 mixture sets generated in Sim2"). Moreover, even though 1000 matrices were generated for both "Orthog" and "Real", only 20 weight matrices with the smallest condition number were used. Results based on those 1000 will be more robust and informative (authors may decide to split the matrices in sets of 100, based on growing condition numbers).

-	From the three simulations (elements in "mix_list", R scripts), it seems like the same cell-type-specific expression profiles are used to both build the mixtures and to be used in the deconvolution. Therefore, this is not realistic and is probably leading to over-optimistic results. Can this be done using half of the samples for the deconvolution and the other half to make the mixtures?

-	GSE64655 contains both heterogeneous mixtures on their own (peripheral blood) and its components (immune cell types). Were all these considered as individual cell types to make mixtures? Since the first one is a linear combination of the constituent cell types this would be a clear case of multi-collinearity.

-	The statements "Library size normalization is required to ensure the deconvolution accuracy", "we observed degraded performance with the count unit and improved performance after library size normalization", "We thus recommend researchers to select an RNA-seq quantification unit that is normalized by library sizes to mitigate the bias" and "Always use quantification unit (countNorm, cpm or tpm) that is normalized by library sizes" are counter-intuitive with respect to "We also noticed the impact of RNA-seq quantification unit is trivial (Supplementary Fig. 3 and 4) and thus selected the most commonly used unit tpm for all remaining illustrations."

-	Regarding the statements appearing on lines 317-319, 240-242, 171-174, 308-310: While I see how a more stable set of solutions (weight matrix with lower condition number) improves the performance, this is never a parameter controllable by researchers (implicit biology in heterogeneous samples) and is actually the output from the deconvolution, so it's unknown in advance.

-	Fig 2e-f + Sup. Fig 8 only shows scatterplots based on values themselves (e.g. lowest vs lowest values on bottom-left corner, but they can be two different genes), but it doesn't keep the gene:gene relationship. Please provide a new version where this relationship is kept (rather than the "agnostic" gene expression magnitude). It will likely show that, even for the case where pt = 0.5 (with sigma = 10, as indicated in #Methods_simulation.R); nb and log-normal still lead to faithful reconstructions whereas "normal" completely distorts the final result.

-	In "#sim2_params_set" 342 samples (combination from 4 datasets) where used but: a) some cell types are present in multiple datasets (e.g. monocytes) while others were only present in one; b) authors pre-fixed which cell types were present in the mixtures of 5 components: ("T", "B", "Mono","Neutro", "NK"); …; 10 components: ("CD4T","CD8T", "NaiveB", "MemoryB","Mono","Neutro","NK", "Eosino","MyeloidDC","HSC"), etc. This simulation should be done allowing all cell types being present in all mixtures (regardless of the number of components) and in more than only 48 conditions (Sup. Table 2).

-	If I understood correctly, the component range is only specified in one R file as follows: Lymphocytes: 14 - 47%; T: 7 - 24; …; HSC: 0.03 - 0.06. and comes from reference 15 ("Inc., S. T. Frequencies of Cell Types in Human Peripheral Blood. (2017)"). I was not able to retrieve such values. Please, provide the original link to such publication and include the proportion values as a table in the manuscript.

-	In Sim1_simModel, the authors found that the noise structure is the main factor obscuring deconvolution performance assessment. While this is not surprising, can the authors provide a "reasonable noise value" that can be found in real mixtures?

-	From sim2_refGenerator.R: alpha = 0.01, nFold = 10 and at least two markers per cell type are required. This is key information that is missing in the manuscript.

-	Related to "Tumor spike-ins were introduced to the 12 mixture sets generated in Sim2". Is this merely addition of counts prior to normalization? Was this done only for markers of the different immune cell types or for all genes?

-	Authors claim that "we observed deconvolution methods performed better on mixtures with smaller tumor content (Fig. 5a, b)". This will also happen with any other cell type that is present in a mixture but not in the reference (see the 2 publications mentioned on the first point).

-	Fig4a-b and Sup. Fig 5a-b show that MMAD and CAMmarker are among the top performers. However, Fig4c clearly shows a bad performance mainly due to a) Neutrophils and Myeloid DCs in MMAD, b) Neutrophils and HSC in CAMmarker. Since the markers used across all methods are the same and Vallania et al., 2018 showed that "different methods have virtually no or minimal effect once the basis matrix is chosen" and MuSiC/CIBERSORT clearly showed the lowest errors (Fig4c, Fig4e, Sup. Fig12, Sup. Fig14), what can be the reason for this astonishing differences?

-	To my knowledge, the statement on lines 179-182 is actually due to the fact that smaller proportions are more difficult to predict accurately. Two new scenarios must be evaluated: a) cases with 5 to 10 components where n-1 cell types have all small proportions and only one (always the same cell type) is big; b) cases with 5 to 10 components with equal proportions across all cell types.

-	Looking at Supp. Fig 14, the statement on lines 211-214 doesn't seem to be valid for several methods and across different number of components. Moreover, on Sup. Fig. 14, why does EPIC start to have very high errors with MyeloidDCs after >= 8 components? Perhaps the additional cell types included are highly correlated with the myeloid DCs?

-	Accordingly to their statement on lines 188-192 (which is also supported in doi.org/10.1101/358366), Pearson correlation figures should be moved from the main manuscript to Supplemental and move the mAD figures from Supplemental to the main manuscript (namely Fig 4a,b; Fig 5a,b; Sup .Fig 15a,b, Sup. Fig 12a,b).

-	Each cell in the different heatmaps indicate averaged correlations/mADs values, which, in my opinion, leads to a loss of relevant information. Median values are more robust than mean values and a better visualization (e.g. boxplots where dots are visible) may provide additional valuable information.

-	The main manuscript mentions "Minimum expression thresholds are set as 10 counts, 1 tpm and 1 fpkm" but looking at the R scripts, different values are used depending on the dataset: dat.filter(…,5,10) for GSE51984, GSE60424 and GSE64655; dat.filter(…,4,10) for GSE113590 and no data filtering for GSE115736 and GSE118490 datasets. Why?

-	I understand why EPIC requires additionally "refProfiles.var" which was made using the IQR but why is there a "cibersort specific ref anno"?

-	Since all datasets used in the manuscript are all bulk RNA-seq datasets, how did the authors use MuSiC? (MuSiC = Multi-subject Single-cell Deconvolution: cell type proportions from bulk sequencing data based on multi-subject single cell data).

-	On Sup. Table 3, MuSiC is labelled as signature based. However, the "music_prop" function has "markers = NULL" as default, meaning that all genes are used.

-	Last paragraph in "Background" belongs to Discussion/Results, and should reflect that CIBERSORT and MuSiC are also best performers with both orthog and real weight matrices (Supplementary Fig. 12).

Comments for the GitHub repository:

-	Please, make a "master" script where scripts are run in order (or at least provide a README with the correct order in which scripts have to be executed).

-	Please provide the output files from RSEM and the associated parameters that were used. I tried to run "sim2_params_set.R" and it did not work because '/mnt/data/haijing/simDeconv/Raw/GSE51984/RSEM_quant/' did not exist.

-	Remove "/mnt/data/haijing/simDeconv/paper_deconvBenchmark" from all paths in your scripts and add a path with respect to the global Github folder.

-	/mnt/data/haijing/simDeconv/Raw/ is missing.

-	Please double-check the R scripts in order to load all required libraries (e.g. GSE113590_varStructure.R has a ggsave but ggplot2/reshape is not previously loaded; error with "scientific_format" because the "scales" R package is not loaded).

-	Please indicate library requirements beforehand (README) on Github (e.g. library(genefilter) in Functions_all.R); clearly indicating which ones available from CRAN, BioConductor, or Github + investigate dependencies local (system) libraries needed (e.g. gfortran/lgfortran)

-	For users that acquire the license to use CIBERSORT or the code for MMAD individually (which I did), please include wrapCIBERSORT.sh; cibersort_oneMat.sh and wrapMMAD ("otherPlatform"), otherwise it will not work.

-	sim3_estW.RData seems to be the output from 2 different scripts (and probably over-written?): s2_sim3_deconvolution_rPlatform.R and s3_sim3_deconvolution_crossPlatform.R

Minor comments:

-	Incomplete line 398: "N is the"

-	Line 16: "studied impacts" should be "studied the impact of"

-	Line 188 from the main refers to Sup. Fig. 12b, which I reckon is missing the "all column" (or perhaps they meant Sup. Fig 14).

-	Sup. Table 2: Factor 3 "Other noise source" not described in summary of factors and Factor 4 "Units" in Sim1_simModel is actually Factor 1 "Quantification units" in Sim1_libSize.

-	Fig. 4c (TIMER), OY axis goes up to 2.1 whereas the rest are smaller than 1 (100%).

-	The authors discarded CAMfree method in Sim2 due to the poor scalability on mixtures with large component number. Since it is also not present in Fig5, I assume it was also discarded in sim3?

-	Please provide a toy example to illustrate the definition of absolute and relative estimation. From the manuscript (lines 231-236) I understand it differently.

**Authors Response**

**Point-by-point responses to the reviewers’ comments:**

The present manuscript describes an in silico framework to benchmark computational methods predicting cell type composition from bulk gene expression measurements. The work explores different in silico models to build the artificial mixtures (different models of noise, different models of weight for each cell type, addition of unknown cell types in the mixture) and tests the performance of different tools.

Major comments.

- All the work is restricted to "in silico" generated mixtures of cell type. While this is convenient since the ground truth is directly know, in silico generated mixtures do not reflect the complexity of biological systems (e.g., the fact that reference gene expression profiles change in an often unknown way when mixed together, the use of biologically unrealistic proportions of cell types, the impact of cell type isolation, the variability between different individuals, the impact of different experimental sequencing procedures, the presence of other unknown cell types than only cancer cells, etc., etc.) . As a result, the best methods identified in this work may just be the ones whose underlying model is closest to the model used to computationally generate the mixtures. This is a major limitation of the work, which prevents reaching solid conclusion about the accuracy of a method in a biologically relevant context.

*We agree with the reviewer that the biological system is complex and challenging to simulate. To systematically study the impacts of several biological and technical factors, we need to generate samples from numerous conditions. However, it is infeasible to build a testing framework covering such a large number of conditions using experimental approaches. Moreover, under a more biologically relevant testing framework, it comes more challenging to interpret benchmark results due to the noise confounding issue in a real dataset. For instance, in a bulk RNA-seq dataset, it is impossible to distinguish noise from the cell-type isolation to the noise from the sequencing procedure. To comprehensively benchmark different deconvolution approaches and help users select appropriate methods, we selected the in silico testing framework to generate large numbers of finely tuned testing environment. Furthermore, our efforts to refine the simulation strategy for deconvolution analysis in this study answer some concerns raised by the reviewer. The reviewer raised concern that "the fact that reference gene expression profiles change in an often unknown way when mixed together." We actually used real RNA-seq profiles from homogeneous sample sources, which means the primary building block of our simulated data is indeed biologically relevant. Artificial signals in a mixture are introduced in the mixing process through noises and predefined proportions. For the noise, to derive a more ‘authentic’ noise structure close to the variance structure in the real data, we tested three types of noise models (log-normal, normal and negative binomial) and selected the negative binomial model as it derives noise structures that are closest to the real data (Figure 2). We plotted a simulated expression profile (y-axis) against the expression profile of a biological mixture (x-axis). As we expected, most of the genes behave concordantly between those two profiles, indicating in silico mixing can recapitulate complexity of the biological mixture (Figure1).*

*Figure 1 Concordance between a simulated mixture vs. a total white blood cell sample (P_X indicates the noise level, r indicates the spearman’s correlation, and d indicates the Euclidean distance)*

*The reviewer raised concern on the 'use of biologically unrealistic proportions of cell types.' We actually generated cellular proportions that mimic PBMC compositions by sampling compositional information defined by the real data from https://www.stemcell.com/media/files/wallchart/WA10006-Frequencies_Cell_Types_Human_Peripheral_Blood.pdf. For example, Monocyte is sampled from a range from 2% to 12%.*

*The reviewer raised concern on “the impact of cell type isolation, the variability between different individuals, and the impact of different experimental sequencing procedures.” The impact of cell type isolation is unavoidable as long as the purification process is involved in the experiment, which means this type of impact affects both in silico testing frameworks and other biologically relevant testing frameworks with coupled FACS or scRNA-seq data. The impact of*

*variance derived from different individuals and experimental sequencing procedures can be captured in the in silico testing framework.*

*The reviewer raised concern on 'the presence of other unknown cell types than only cancer*

*cells.' Although this simulation framework does not evaluate unknown non-cancer cell types, it*

*provided a reasonable estimate in which unknown cell types could be finely tuned and tested. Moreover, creating an experimental dataset with coupled unknown cell-type proportions is still challenging as these unknown contents are still not well studied.*

*We agree with the reviewer's point that the best methods identified in this work may be the ones whose underlying model used is closest to the simulated mixtures. However, this is a limitation applies to both in silico frameworks and more biologically relevant frameworks. For example, most of the benchmark data with coupled FACS results only contain a limited number of samples and cellular components. Moreover, the number of these biologically relevant benchmark dataset is also limited. Therefore, we cannot guarantee the methods perform well under one biological context can stand out under other biological contexts. On the other hand, simulation can mitigate the robustness issue by increasing the throughput, determining outperforming methods according to the tested condition. For instance, MMAD and DSA showed good performance in the Sim1, CIBERSORT and MuSiC performed well with 'real' compositions in the Sim2, and EPICabsolute performed well in the absolute scale in the Sim3.*

- The authors claim that benchmarking studies are lacking. However, this is not true. First several studies where new methods were developed spent time to find a good framework to robustly validate the results in a biologically relevant context (i.e., not only with artificial mixtures but for instance using single-cell RNA-Seq data, or samples analyzed by flow cytometry or by immuno-histochemistry in parallel to gene expression). Second, several benchmarking studies have been recently published (e.g., Sturm et al Bioinformatics 2019), and many of them are just not cited.

*As the reviewer pointed out, most deconvolution method paper do carry out a benchmark to claim that the newly developed method is better. However, due to inconsistent testing frameworks, it is difficult for researchers to reach a consensus conclusion and to determine the optimal method for their deconvolution analysis. To provide a reliable reference for the application and development of deconvolution methods, we need to establish benchmarking frameworks that mimic application scenarios of more complicated and diverse biological systems. We thank the review for pointing out the study by Sturm et al. 2019. Sturm only compared six reference-based deconvolution methods while we evaluated 11 methods spanning three categories: marker-based, reference-based and reference-free methods. Major conclusions of Sturm's benchmarking study heavily relied on the in silico testing frameworks where they constructed pseudo-bulk tissue by aggregating scRNA-seq profiles. More biologically relevant benchmark RNA-seq data couples with composition data from FACS, IHC and scRNASeq data is still severely restricted by the sample number and cellular component number. As we can see from Sturm’s paper, where they collected 3 biologically relevant benchmark datasets: Hoek (n =8), Racle (n = 4), and Schelker (n=3) (n is the sample number). Above sample number scale (n =15) is unable to derive benchmark study that can be used learn joint impact of different biological and technical factors. Another issue is the inconsistent cell-type annotation makes it very difficult to compare assessment results derived from different biologically relevant benchmark datasets. For instance, while Hoek data profiled T cell frequencies in the analysis, Racle and Schelker's data profiled CD4+ and CD8+ T cell frequencies. In our study, one simulated dataset contains 20 samples, and we have built more than 1,766 conditions; therefore, we tested 35,320 samples in total. We have now cited Sturm's paper in the Background (Page 3) and Discussion Sections (Page 18) in our manuscript.*

- Throughout the manuscript, it is very difficult to know what kind of cell types are used to build the mixtures, or for other analyses. One example (but this is not the only one) is line 106-109, where there is no indication about what the 'real' data are, and why this analysis enables to discriminate different noise models. Another example is the addition of tumor spike-ins, where I could not find what type of tumor samples have been used (are the authors even sure that these were profiles from pure cancer cells, without cells from the micro-environment?). These issues highlight that the work is mainly computational with some relevant algorithmic/methodological observations, but often quite disconnected from the real issues with biological samples.

*106-109 is the summary of the variance analysis, the detailed explanation is in 110-143 of our original manuscript (145-179 of our revised manuscript).*

*We apologize for the confusion. We now added the cell-type identity of real data in the Figure 2 For more detailed information of variance structure analysis of real data, please refer to Supplementary Figure 6 and 7. For the unknown cellular contents, we used HCT116 samples, which is human colon cancer cell line. This piece of information is in the Supplementary Table 2 and Dataset Description Section in the original manuscript. We selected this cell type because it has a very distinct profile to the immune components so that we can avoid noise from unwanted contamination. We have now attached a new table (Supplementary Table 4) to list every cellular component used in all analysis.*

- Some cell types are easy to 'deconvolve' (e.g., major immune cell types), while others are much more difficult. This is a major issue in cell type deconvolution, which is almost totally overlooked in the present manuscript.

*We have now added our discussion on closely related cell types in the Result and Discussion section in the revised manuscript. (Page 12 Line 262 – 265, Page 18 Line 281)*

- Many observations (lower performance with increased noise, with 'real' compared to 'ortholog' weight matrices, with increased number of components) are quite expected and well known in the field and in previous studies.

*It is not difficult to conclude that with a larger noise level and more component numbers, an algorithm's performance would degrade. This conclusion not only holds for deconvolution analysis but also for other types of data analysis. However, this benchmark paper focuses on the tipping point where each method starts to deteriorate. The value of benchmarking is to figure out the performance boundaries of each method quantitatively.*

Other comments:

- CIBSERSORTx should be included in the benchmark.

*We have now added CIBERSORTx in the revision. Overall, CIBERSORTx and CIBERSORT exhibit very similar performance in two evaluation metrics (correlation and mAD) used in this study (CIBERSORTx is now added to all Figures & Results).*

- The authors should think whether the word 'deconvolution' is really the best. Strictly speaking, deconvolution means predicting not only the fraction of the different cell types, but also the actual gene expression profile for each cell type (something much mode difficult). I'm aware that deconvolution is often used for predicting cell type composition, but maybe worth clarifying this point.

*We agree with the reviewer that deconvolution has a broader definition than cell type proportion estimation. Unfortunately, the word ‘deconvolution’ has already been adopted by many manuscripts in our field indicating estimation of compositional information. We’ve now clarified this point in the revised manuscript (Line 384 - 388).*

- Line 305: this is actually not best practice in deconvolution analysis, but in building in silico benchmarks for testing deconvolution tools. Quite a different problem…

*We agree with reviewer’s comment. We have now changed our wording in Discussion Section (Page 18, Line360 - 379).*

- It is not always clear how different tools were used. For instance, several tools cannot distinguish memory B cells from naive B cells, but results are still reported for these cell types in Figure 4 for all the tools.

*For all methods, we used the default parameter settings. The detailed parameters for applying each method are in our scripts, which is publicly available at Github repository https://github.com/LiuzLab/paper_deconvBenchmark. When a deconvolution method returned NA value, we assign a penalty value (correlation: -1 and mAD: 1) to ensure the failure is reflective in the final evaluation metrics (Method section, line 532-534).*

Reviewer 2:

-	It is not clear, however, whether they are aware of two highly related studies that have recently appeared: "Comprehensive evaluation of transcriptome-based cell-type quantification methods for immuno-oncology" and "Comprehensive benchmarking of computational deconvolution of transcriptomics data", which cover topics quite similar to the present work. Therefore, I recommend that the authors revise their text to comprehensively relate their discussion to the prior work by others to more clearly indicate what new insights arise from their study.

*Thank you very much for pointing out these two relevant studies (Sturm et al. Bioinformatics 2019 and Cobos et al. bioRxiv 2020). Sturm et al. focused on spill-over effects, minimal detection fraction, and background predictions and suggested removing non-specific signature genes to improve deconvolution accuracy. Cobos et al. focused on the impacts of different normalization strategies, marker gene selection strategies, and missing cellular components in the reference. Our study focused on the impacts of simulation model selection, noise levels, quantification unit selection, cellular component number, weight matrix property, and unknown cellular contents. In our opinion, all three studies have very different focuses and provided valuable information to the field of deconvolution. Compared with previous benchmarks, we applied more flexible simulation strategies to create mixtures covering dynamic conditions, enabling us to investigate the tipping point where each method deteriorates. Moreover, we studied the impact of currently used simulation strategies and suggested new strategies of deriving better benchmarks such as using the negative binomial model to recapitulate noise structure in the real data, referencing proportions in the real data, and separating evaluation based on the absolute scale and relative scale when unknown cellular content exists. We have now included the review of these two benchmark studies to the Background (Page 3) and Discussion section (Page 18).*

-	With the exception of "Sim1_simModel", I find the number of mixtures evaluated in the different simulations to be quite limited to produce robust conclusions (e.g. "Tumor spike-ins were introduced to 12 mixture sets generated in Sim2"). Moreover, even though 1000 matrices were generated for both "Orthog" and "Real", only 20 weight matrices with the smallest condition number were used. Results based on those 1000 will be more robust and informative (authors may decide to split the matrices in sets of 100, based on growing condition numbers).

*We apologize for the error and have now modified the description in our revised Manuscript (Line 509-510).We selected the matrix that has the smallest condition number over 1,000 matrices from the uniform distribution. This filtering procedure is necessary to ensure the orthogonality of the weight matrix. We observed that most of the algorithm performs better in the ‘orthog’ condition compared to ‘real’ condition (Fig 4a,b and Fig 5a,b) . Creating 10 sets of 'orthog' weight matrices would likely lead to the same conclusion.*

-	From the three simulations (elements in "mix_list", R scripts), it seems like the same cell-type-specific expression profiles are used to both build the mixtures and to be used in the deconvolution. Therefore, this is not realistic and is probably leading to over-optimistic results. Can this be done using half of the samples for the deconvolution and the other half to make the mixtures?

*In our first simulation framework (Sim1_simModel and Sim1_libSize), 3 sets of independent mixtures and references are generated (Supplementary Figure 1). In the following deconvolution analysis, we used all possible mixture – reference pairs and averaged our results. For one testing environment, there are 9 (3 times 3) deconvolution results from which 6 of them have mixture- reference pairs derived from different sources. For simplicity, we only presented the averaged performance across 9 mixture-reference pairs, but the impact of mixture-reference variance is considered in this analysis (Line 474 - 482).*

-	GSE64655 contains both heterogeneous mixtures on their own (peripheral blood) and its components (immune cell types). Were all these considered as individual cell types to make mixtures? Since the first one is a linear combination of the constituent cell types this would be a clear case of multi-collinearity.

*All simulated mixtures started from purified homogeneous profiles. When we were creating references, we only picked those samples derived from homogeneous sources. We avoided using heterogeneous samples, typically named by ‘whole blood’, ‘whole white blood', or ‘pbmc’, for reference generation. Those heterogeneous samples are only used in the variance analysis to deduce the upper boundary of noise in the real data.*

-	The statements "Library size normalization is required to ensure the deconvolution accuracy", "we observed degraded performance with the count unit and improved performance after library size normalization", "We thus recommend researchers to select an RNA-seq quantification unit that is normalized by library sizes to mitigate the bias" and "Always use quantification unit (countNorm, cpm or tpm) that is normalized by library sizes" are counter-intuitive with respect to "We also noticed the impact of RNA-seq quantification unit is trivial (Supplementary Fig. 3 and 4) and thus selected the most commonly used unit tpm for all remaining illustrations."

*The library size normalization has trivial impacts on the Sim1_simModel because samples in Sim1_simModel have the same library size. To reveal bias caused by varied library sizes, we designed Sim1_libSize in which every mixture comprised of samples with varied library sizes (first 10 samples with 12M reads, and remaining 10 samples with 24M reads) and our results indicate using quantification units normalized by library sizes can mitigate the bias mentioned above (Line 122-126, Line 192 – 194).*

-	Regarding the statements appearing on lines 317-319, 240-242, 171-174, 308-310: While I see how a more stable set of solutions (weight matrix with lower condition number) improves the performance, this is never a parameter controllable by researchers (implicit biology in heterogeneous samples) and is actually the output from the deconvolution, so it's unknown in advance.

*While we agree that the weight matrix is not controllable by the user, researchers can roughly speculate some basic properties of the weight matrix from previous studies. For instance, cancer samples tend to be more orthogonal than healthy tissues due to the disrupted tissue structure. Researchers generally have a rough idea about the level of unknown cell types or minor cellular components in the tissue they study. These pieces of prior information will help them to decide the appropriate tool to derive more accurate estimates.*

-	Fig 2e-f + Sup. Fig 8 only shows scatterplots based on values themselves (e.g. lowest vs lowest values on bottom-left corner, but they can be two different genes), but it doesn't keep the gene:gene relationship. Please provide a new version where this relationship is kept (rather than the "agnostic" gene expression magnitude). It will likely show that, even for the case where pt = 0.5 (with sigma = 10, as indicated in #Methods_simulation.R); nb and log-normal still lead to faithful reconstructions whereas "normal" completely distorts the final result.

*Dots in Figure 2e-f and Supplementary Figure 8 are actually plotted on the same gene since the expression is not averaged or binned. Each dot in the scatter plots represent one gene in two samples (the x-axis and y-axis). The gene-gene relationship is kept. If the gene expression levels are similar in two samples, the dot will be closer to the diagonal line, which indicates y = x.*

*For the scenario where pt=0.5, sigma is 10, we observed that only nb models lead to a faithful reconstruction whereas ‘log-normal’ and ‘normal’ completely distorts the variance structure between samples (Figure 2e and f). We also observed the same trend in all other parameter settings (Supplementary Figure 8).*

-	In "#sim2_params_set" 342 samples (combination from 4 datasets) where used but: a) some cell types are present in multiple datasets (e.g. monocytes) while others were only present in one; b) authors pre-fixed which cell types were present in the mixtures of 5 components: ("T", "B", "Mono","Neutro", "NK"); …; 10 components: ("CD4T","CD8T", "NaiveB", "MemoryB","Mono","Neutro","NK", "Eosino","MyeloidDC","HSC"), etc. This simulation should be done allowing all cell types being present in all mixtures (regardless of the number of components) and in more than only 48 conditions (Sup. Table 2).

*a) We are fully aware that some cellular components' profiles are derived from several datasets,while others are only present in one. This is due to the lack of cellular components per dataset, so we had to concatenate samples from 4 datasets to increase the number of usable cellular components in Sim2.*

*b) Sim2 simulation is intended to show the impacts of the increasing component number to the deconvolution analysis. Thus, we generated gradients of component numbers from 6 to 10 to show deconvolution algorithms' tipping point when the component number is increasing. Pre- fixing cell types in each mixture do serve the need for the purpose of Sim2.*

-	If I understood correctly, the component range is only specified in one R file as follows: Lymphocytes: 14 - 47%; T: 7 - 24; …; HSC: 0.03 - 0.06. and comes from reference 15 ("Inc., S. T. Frequencies of Cell Types in Human Peripheral Blood. (2017)"). I was not able to retrieve such values. Please, provide the original link to such publication and include the proportion values as a table in the manuscript.

*The original link is: https://www.stemcell.com/media/files/wallchart/WA10006-Frequencies_Cell_Types_Human_Peripheral_Blood.pdf. We have now attached the link to the reference (Line 630-632).*

-	In Sim1_simModel, the authors found that the noise structure is the main factor obscuring deconvolution performance assessment. While this is not surprising, can the authors provide a "reasonable noise value" that can be found in real mixtures?

*For PBMC data, we consider the variance between whole blood samples as the upper limit of noise level since heterogeneous samples contain both biological variance and compositional variance. According to our variance analysis results in Supplementary Figure 6 and 7, spearman’s correlation (all genes) around 0.5 and Euclidean distance in the order of 10! and 10" is considered as the normal range for the PBMC samples. However, these two metrics can vary according to the sequencing platforms, sample quality, and tissue type. Therefore, we recommend users to derive the boundary of noise from their own bulk samples based on above two metrics.*

-	From sim2_refGenerator.R: alpha = 0.01, nFold = 10 and at least two markers per cell type are required. This is key information that is missing in the manuscript.

*Thank for reminding us about this. We have now added this piece of information in the method section of manuscript (Page 20, Line 415-428).*

-	Related to "Tumor spike-ins were introduced to the 12 mixture sets generated in Sim2". Is this merely addition of counts prior to normalization? Was this done only for markers of the different immune cell types or for all genes?

*The addition of counts was carried out before normalization on all genes. We have now added this clarification to the method section (Page 25, Line 523-524).*

-	Authors claim that "we observed deconvolution methods performed better on mixtures with smaller tumor content (Fig. 5a, b)". This will also happen with any other cell type that is present in a mixture but not in the reference (see the 2 publications mentioned on the first point).

*We agree with the reviewer. Although a tumor profile is used as an unknown component in our analysis, the conclusion can be generalized to any cell types that are not included in the reference profiles.*

-	Fig4a-b and Sup. Fig 5a-b show that MMAD and CAMmarker are among the top performers. However, Fig4c clearly shows a bad performance mainly due to a) Neutrophils and Myeloid DCs in MMAD, b) Neutrophils and HSC in CAMmarker. Since the markers used across all methods are the same and Vallania et al., 2018 showed that "different methods have virtually no or minimal effect once the basis matrix is chosen" and MuSiC/CIBERSORT clearly showed the lowest errors (Fig4c, Fig4e, Sup. Fig12, Sup. Fig14), what can be the reason for this astonishing differences?

*Firstly, most figures in Vallania et al. used indirect assessment such as goodness of fit (correlation between true mixtures vs. reconstructed mixtures) and correlation of estimations derived from different methods. In other words, they did not perform a direct assessment of deconvolution methods that compare estimated proportions to the ground truth. Secondly, the only result involved the direct assessment is Figure 4 in Vallania et al. They used RMSE and Pearson’s correlation coefficients to compare deconvolution estimations to the FACS data (ground truth).*

*• In Figure 4, they mainly discussed the impact of using different signature matrices. Therefore, they concluded that the selection of signature matrices is crucial for deconvolution analysis.*

*• While I agree with part of their conclusion about the signature matrix, they obviously overlooked the impact of mixture sample condition. For instance, in Figure 4a of Vallinia et al., if we look at the LM22 lane, we can find a huge variation of CIBERSORT’s accuracy on different datasets (The correlation varied from 0.25 – 0.8). This phenomenon is consistent with our study's conclusions that the condition of mixture samples significantly impacts deconvolution accuracy.*

*In summary, we agree with Vallina et al. that the signature matrix will impact the outcome of deconvolution while our paper focuses on the impact of the mixture condition, which can help explain the differences raised by the reviewer.*

-	To my knowledge, the statement on lines 179-182 is actually due to the fact that smaller proportions are more difficult to predict accurately. Two new scenarios must be evaluated: a) cases with 5 to 10 components where n-1 cell types have all small proportions and only one (always the same cell type) is big; b) cases with 5 to 10 components with equal proportions across all cell types.

*We implemented the analysis requested by the reviewer.*

*• Indeed, we observed that reference-based methods like CIBRSORT and MuSIC tend to work well in both scenarios (referred to as a and b in the Figure shown below), while marker-based and reference-free methods failed in the analysis.*

*• In our opinion, minor cellular components and highly correlated cellular components are byproducts of increasing component numbers. Our study did not further decompose these two factors since these two factors are often confounded and require a different testing framework to investigate. We have now modified our statement in the revised manuscript (Line 261-265).*

*Figure 2 Scatter plots of estimated proportions to the ground truth (x-axis: truth, y-axis: estimation, a refers to the a scenario, b refers to the b scenario)*

-	Looking at Supp. Fig 14, the statement on lines 211-214 doesn't seem to be valid for several methods and across different number of components. Moreover, on Sup. Fig. 14, why does EPIC start to have very high errors with MyeloidDCs after >= 8 components? Perhaps the additional cell types included are highly correlated with the myeloid DCs?

*Supp. Fig 13 is based on the correlation metric and Supp Fig. 14 is based on the mean absolute deviance. Supp Fig.14 is not enough to derive conclusions about estimation accuracy, since the baseline mean absolute deviance of the MyeloidDC (<1%) is low and might appeared as high accuracy. However, when we look at the correlation metrics in Supp Fig. 13, all methods failed to derive high correlation value due to the poor concordance of estimations to the ground truth. Taken together both figures, we concluded that none of the methods we studied performed well on cell types with extremely small proportions.*

-	Accordingly to their statement on lines 188-192 (which is also supported in doi.org/10.1101/358366), Pearson correlation figures should be moved from the main manuscript to Supplemental and move the mAD figures from Supplemental to the main manuscript (namely Fig 4a,b; Fig 5a,b; Sup .Fig 15a,b, Sup. Fig 12a,b).

*The reviewer is right. We found out that the degraded performance is due to the multicollinearity issue which is caused by signature genes of HSC components. We calculated the correlation of before (comp7_cor) and after (comp8_cor) introducing HSCs subsetted by signature genes used by EPIC and found out from Comp8 different levels of multicollinearity effects exacerbated. We looked at the EPIC method paper(Racle et al. 2017) and didn’t find any regularization term to mitigate collinearity of reference profiles.*

*We agree with the reviewer that using correlations has its limits. However, correlation is still regarded as the primary evaluation metric due to its capability of revealing the concordance between ground truth and estimation. Only looking at mAD value cannot determine the concordance between estimates and truth. In summary, we believe the two metrics need to be considered together.*

-	Each cell in the different heatmaps indicate averaged correlations/mADs values, which, in my opinion, leads to a loss of relevant information. Median values are more robust than mean values and a better visualization (e.g. boxplots where dots are visible) may provide additional valuable information.

*We chose mAD (mean absolute deviance) values to make our results comparable to previous studies as averaged deviations are the most commonly used supplementary evaluation metrics. Sturm et al.(Sturm et al. 2019), Shelker et al.(Schelker et al. 2017), and Cobos et al. (Cobos et al. 2020)used Root mean square deviation (RMSD). Wang et al.(Wang et al. n.d.) used Mean absolute deviance(mAD).*

*We draw boxplots of absolute deviance from Sim2 Comp 10 mixture (real weight matrix, tpm unit) to show differences between mean absolute deviance and median absolute deviance. In this figure, we observed that the mean and median of absolute deviance are very close to each other, and the same conclusion will be reached regardless of the metric’s selection.*

*Figure 3 Box Plot of Absolute Devicances of Sim2 Real Comp10 Mixture (Red dot – mean, Line – median; Unit - TPM*

-	The main manuscript mentions "Minimum expression thresholds are set as 10 counts, 1 tpm and 1 fpkm" but looking at the R scripts, different values are used depending on the dataset: dat.filter(…,5,10) for GSE51984, GSE60424 and GSE64655; dat.filter(…,4,10) for GSE113590 and no data filtering for GSE115736 and GSE118490 datasets. Why?

*• The parameter (..., 5, 10) is used to retain genes with more than 10 counts in at least 5 samples. We modify this parameter slightly according to the available sample numbers.*

*• In the Sim1, we performed filtering independently on each dataset with the minimum sample thresholding set at 5. For Sim2 and Sim3, we first concatenated samples into one matrix and then performed filtering with minimum sample threshold set at 10. For the involved datasets of Sim1, Sim2, and Sim3, please refer to the Supplementary Table 4.*

*• GSE113590 only has 4 samples per cellular category, thus set the parameter to 5 is impossible.*

*• For GSE115736 and GSE118490, we performed filtering in a different script (sim2_params_set.R and sim3_params_set.R). We have now added theses technical details in the Data_description.txt file under the root directory of the Github Repository and Methods section (Line 405 - 414).*

-	I understand why EPIC requires additionally "refProfiles.var" which was made using the IQR but why is there a "cibersort specific ref anno"?

*CIBERSORT software requires an annotation file containing the reference's cell-type identity information. "cibersort specific ref anno" is indeed the required annotation file for the CIBERSORT software.*

-	Since all datasets used in the manuscript are all bulk RNA-seq datasets, how did the authors use MuSiC? (MuSiC = Multi-subject Single-cell Deconvolution: cell type proportions from bulk sequencing data based on multi-subject single cell data).

*MuSiC can use bulk RNA-seq data as reference and is actually one of the top-performing methods in our study.*

-	On Sup. Table 3, MuSiC is labelled as signature based. However, the "music_prop" function has "markers = NULL" as default, meaning that all genes are used.

*MuSiC does not rely on the preselected marker genes or signature genes since it implements weighted non-negative least squares regression (W-NNLS). We did not use any pre-determined genes, as recommended in the author's manual (https://xuranw.github.io/MuSiC/articles/MuSiC.html). To avoid confusion, we transformed the term 'signature-based' to the 'reference-based' in our manuscript (Line 100 - 103).*

-	Last paragraph in "Background" belongs to Discussion/Results, and should reflect that CIBERSORT and MuSiC are also best performers with both orthog and real weight matrices (Supplementary Fig. 12).

*We have now fixed the wording (Line 351 – 352).*

Comments for the GitHub repository:

-	Please, make a "master" script where scripts are run in order (or at least provide a README with the correct order in which scripts have to be executed).

*A README file has been added to the root directory to indicate the execution order of the scripts.*

-	Please provide the output files from RSEM and the associated parameters that were used. I tried to run "sim2_params_set.R" and it did not work because '/mnt/data/haijing/simDeconv/Raw/GSE51984/RSEM_quant/' did not exist.

*• I have attached the compressed Raw.tar.gz file to the google drive.*

*• The file includes all RSEM outputs with the folder structure matched to the analysis scripts.*

*• We also add RSEM associated parameters in our Method section (Line 403).*

-	Remove "/mnt/data/haijing/simDeconv/paper_deconvBenchmark" from all paths in your scripts and add a path with respect to the global Github folder.

*We agree with the reviewer that we should avoid using absolute path whenever possible. Nevertheless, we used the absolute path to avoid encountering technical issues for non-R- platform-based methods like MMAD, CIBERSORT, and CIBERSORTx, where we need to trigger wrapper scripts (.sh or .m files) through the system command. We have now highlighted this issue in relevant scripts and add comments suggesting users modify the path parameter according to their working directory.*

-	/mnt/data/haijing/simDeconv/Raw/ is missing.

*The Raw data is uploaded to the Google drive.*

-	Please double-check the R scripts in order to load all required libraries (e.g. GSE113590_varStructure.R has a ggsave but ggplot2/reshape is not previously loaded; error with "scientific_format" because the "scales" R package is not loaded).

*The ‘scales’ library is indeed loaded in src/Methods_evaluation.R line 4. I have now added a libraries.txt file that listed all required R packages under the root directory of the Github Repository.*

-	Please indicate library requirements beforehand (README) on Github (e.g. library(genefilter) in Functions_all.R); clearly indicating which ones available from CRAN, BioConductor, or Github + investigate dependencies local (system) libraries needed (e.g. gfortran/lgfortran)

*This piece of information is now attached in the libraries.txt file under the root directory.*

-	For users that acquire the license to use CIBERSORT or the code for MMAD individually (which I did), please include wrapCIBERSORT.sh; cibersort_oneMat.sh and wrapMMAD ("otherPlatform"), otherwise it will not work.

*• For CIBERSORT and CIBERSORTx, users can request for the source code of software from https://cibersort.stanford.edu/ and https://cibersortx.stanford.edu/. After installing the software, they can use the scripts we provided to run the analysis.*

*• For MMAD, users can directly download the software from https://sourceforge.net/projects/mmad/ . To run the dataset on a large scale, please modify the MATLAB code to force the output in .txt file format.*

*• We have now uploaded all the wrapper scripts for CIBERSORT, CIBERSORTx and MMAD to the Github repository (folder: ./code/src/otherPlatform/).*

-	sim3_estW.RData seems to be the output from 2 different scripts (and probably over-written?): s2_sim3_deconvolution_rPlatform.R and s3_sim3_deconvolution_crossPlatform.R

*Yes, sim3_estW.RData contains deconvolution results from all deconvolution methods. s2_sim3_deconvolution_rPlatform.R is the script for R platform based methods and s3_sim3_deconvolution_crossPlatform.R is the script for methods based on other platforms such as MATLAB (MMAD) , JAVA (CIBERSORT) and DOCKER (CIBERSORTx). Therefore, sim3_estW.RData is the output from two scripts.*

Minor comments:

-	Incomplete line 398: "N is the"

*“N is the” has been discarded in the manuscript.*

-	Line 16: "studied impacts" should be "studied the impact of"

*Thank you for the kind remind. We have already fixed the grammar error.*

-	Line 188 from the main refers to Sup. Fig. 12b, which I reckon is missing the "all column" (or perhaps they meant Sup. Fig 14).

*Supplementary Figure 14 is the heatmap of cell-type-specific mAD values. In each heatmap, the last column indicates the averaged mAD across all cell types (row average). Supplementary Figure 12b is the summarized version of Supplementary Figure 14, and each cell in the heatmap indicates averaged mAD values across all cell types in a mixture, which is exactly the last column of every heatmap in the Supplementary Figure 14. (Supplementary Figure 12b doesn’t contain ‘all’ column, since it is exactly the collection of the last column in Supplementary Figure 14)*

-	Sup. Table 2: Factor 3 "Other noise source" not described in summary of factors and Factor 4 "Units" in Sim1_simModel is actually Factor 1 "Quantification units" in Sim1_libSize.

*Other noise source is the noise introduced by reference vs. mixture variance. We have now added the description of the other noise source in both Supplementary Table 2 and in the line 474– 482 of Method section.*

*We have unified the name to the “Unit” (Supplementary Table 2).*

-	Fig. 4c (TIMER), OY axis goes up to 2.1 whereas the rest are smaller than 1 (100%).

*TIMER doesn’t implement sum-to-1 restriction. This is why the Y-axis in Fig. 4c of TIMER goes up to 2.1.*

-	The authors discarded CAMfree method in Sim2 due to the poor scalability on mixtures with large component number. Since it is also not present in Fig5, I assume it was also discarded in sim3?

*Yes, CAMfree showed poor scalability with mixtures contain large cellular components, and we excluded this method in both Sim2 and Sim3.*

-	Please provide a toy example to illustrate the definition of absolute and relative estimation. From the manuscript (lines 231-236) I understand it differently.

*Sorry for the confusion. We have attached following toy example in the Supplementary Table 5 of our revised manuscript.*

*References:*

*Cobos, Francisco Avila, José Alquicira-Hernandez, Joseph Powell, Pieter Mestdagh, and Katleen De Preter. 2020. “Comprehensive Benchmarking of Computational Deconvolution of Transcriptomics Data.”*

*Racle, Julien, Kaat de Jonge, Petra Baumgaertner, Daniel E. Speiser, and David Gfeller. 2017. “Simultaneous Enumeration of Cancer and Immune Cell Types from Bulk Tumor Gene Expression Data.” ELife 6:1–25.*

*Schelker, Max, Sonia Feau, Jinyan Du, Nav Ranu, Edda Klipp, Gavin MacBeath, Birgit Schoeberl, and Andreas Raue. 2017. “Estimation of Immune Cell Content in Tumour Tissue Using Single-Cell RNA-Seq Data.” Nature Communications 8(1):2032.*

*Sturm, Gregor, Francesca Finotello, Florent Petitprez, Jitao David Zhang, Jan Baumbach, Wolf H. Fridman, Markus List, and Tatsiana Aneichyk. 2019. “Comprehensive Evaluation of Transcriptome-Based Cell-Type Quantification Methods for Immuno-Oncology.” Bioinformatics 35(14):i436–45.*

*Wang, Xuran, Jihwan Park, Katalin Susztak, and Nancy R. Zhang. n.d. “Bulk Tissue Cell Type Deconvolution with Multi-Subject Single-Cell Expression Reference.” Nature Communications (2019).*

**Second round of review**

**Reviewer 1**

I appreciate the efforts done by the authors to optimize their in silico framework. However, I'm still very concerned about the lack of biologically relevant benchmark. In particular, throughout the manuscript, I could not find any benchmark that corresponds to a real sitatution faced in biology where one takes a real tissue (i.e. not PBMC) and tries to predict cell type composition. This is an important concern for a paper claiming to perform a "comprehensive benchmark". I'm surprised that the authors did not attempt to address this limitation of their work, especially considering that such data are available, especially with the hundreds of single-cell RNA-Seq datasets, and I'm afraid that their work remains disconnected from the real biological questions that are faced by people applying deconvolution algorithms.

1) "The reviewer raised concern that "the fact that reference gene expression profiles change in an often unknown way when mixed together." We actually used real RNA-seq profiles from homogeneous sample sources, which means the primary building block of our simulated data is indeed biologically relevant".

I do understand that reference profiles cam from biological samples (actually PBMC for immune cells and cancer cell lines for cancer cells), but the issue is that when you mix cells, their expression can change in an often completely unknown way, which is not accurately captured by models of gene expression noise. This is why in silico mixtures fail to recapitulate a very important part of the complexity of real biological samples. Typically, modelling a tissue gene expression profile (e.g., tumor) as a mixture of reference profiles from immune cells from PBMC and from cancer cell lines is just wrong, because it does not integrate the fact that both immune and cancer cells have very different profiles in a real tumor. The same applies to pretty much any solid tissue where deconvolution is practically interesting to perform. This is also why deconvolution works well in PBMC (but is not very useful because it's really easy to measure cell fractions there, even easier than gene expression profiling), but not in other tissues. Not considering this issue results in the fact that only very theoretical conclusions can be drawn from such a benchmark...

2) "For instance, while Hoek data profiled T cell frequencies in the analysis, Racle and Schelker's data profiled CD4+ and CD8+ T cell frequencies. In our study, one simulated dataset contains 20 samples, and we have built more than 1,766 conditions; therefore, we tested 35,320 samples in total." I'm afraid that it is more useful to have a few biologically relevant cases, than thousands of artificial cases which do not capture the complexity of biological samples.

3) "When a deconvolution method returned NA value, we assign a penalty value (correlation: -1 and mAD: 1) to ensure the failure is reflective in the final evaluation metrics (Method section, line 532-534)". Does this mean that simply because a method does not incorporate a given cell type, it gets penalized? This is an important issue, because it could very well be that some methods perform badly simply because they do not incroprate some of the cell types that have been a priori selected by the authors (and this choice is indeed questionable - for instance why separating Memory and Naive B cells, but not the subtypes of CD4 cells?). For instance, many methods do not include predictions for oesinophil, a relatively rare cell type, or a distinction between Naive an Memory B cells, and will therefore be strongly penalized. Also, I do not understand how values have been computed in Figure 4E, since it looks like all methods could be applied to all cell types, which is clearly not the case when using methods with default parameters. Or did the authors retrain the algorithms with new profiles, as some methods enable to include user-defined profiles. This is all very confusing, and I'm afraid the authors need to check more precisely how they used the different methods and provide better explanations in the method section.

Minor comment:

- Regarding the use of the different dataset, it is stil difficult, when reading the result section, to follow which cell type have been used in which cases. I think this could be easily improved by more systematically refering to the dataset number at page 29. Typically line 114-118, or line 200 - 208, where I just could not figure out what data was used (is it the same PBMC as before?), and line 272 - 276, where I do not know where the spike-ins come from. I understand that the background of the authors is more on the algorithmic aspects of building the in silico benchmark, but the choice of datasets has a big importance for benchmarking deconvolution. Putting this information in Figure legend would also help.

- Legend of Figure 2: a, Heatmap of summarized evaluation results based on the Pearson’s correlation coefficients and corresponding b... Looks like something is missing

**Reviewer 2**

Jin and Liu have answered many of my previous remarks and have modified their manuscript accordingly. While the new version already provides a stronger message, there are still a few key aspects that need to be answered (mostly answers they already provided but still haven’t included in the manuscript).

- Primary comments:

* Based on their answer to my first primary comment from the first round of peer-review, Line 14 from the abstract (“due to lack of comprehensive benchmarks”) should be removed because it is not true. Moreover, lines 38-39 (“tested on inconsistent frameworks”) still should be further explained.

* Their answer to my previous question “Were all these considered as individual cell types to make mixtures?” was not included anywhere in the manuscript (“We avoided using

heterogeneous samples, typically named by ‘whole blood’, ‘whole white blood', or ‘pbmc’, for reference generation”).

* Line 122 (“impact of RNA-seq quantification is trivial”) still misses the information provided by the authors in one of their answers “The library size normalization has trivial impacts on the Sim1_simModel because samples in Sim1_simModel have the same library size.” (they also observed it is not a trivial choice in their other simulations: lines 192-194).

* Their new additions in grey throughout the manuscript require extensive English corrections (e.g. “Further studies are still in need decompose the influence of multicollinearity

issue and rare components” / “we explored the impacts of noise structure” / “More benchmarks derive accuracies of cell-type specific expression estimation is still in need” / “we relaxed the criteria by the controlling the percentages of sample number passing the expression threshold”… to name a few).

* On lines 532-534 authors have added “When a deconvolution returns NA values, we directly assign highest penalty for the evaluation metrics: r = -1, and mAD = 1.” Why would a deconvolution method return NA values? Which ones did? This is very relevant information that should be reported.

* Authors should still include in the manuscript their answer “While (we agree that) the weight matrix is not controllable by the user, researchers can roughly speculate some basic properties of the weight matrix from previous studies” when stating it as the most important factor affecting the deconvolution: Lines 289-291, 392-393 and Line 499. This is a key message that needs specific context to be correctly understood.

* Supplementary Table 4 (“Gradient of Cell Types” tab) has to be included in the main manuscript in order to grasp how Sim2 was done (e.g. inside section “Benchmarking framework construction” before “Weight simulations”) .

* Authors did implement 2 very relevant analyses I requested (a) cases with 5 to 10 components where n-1 cell types have all small proportions and only one (always the same cell type) is big; b) cases with 5 to 10 components with equal proportions across all cell types). However, these are neither included in the manuscript nor in Supplemental (or at least not in the PDF version I got, which was missing SFig 1, 2, 6, 8, 9 and 10).

- Comments to the Github repository:

* Authors have also improved their Github repository and provided data from a Google drive link. However: 1) this is not ideal for long-term storage so please upload it to Figshare or similar. 2) the code hasn't been fully adapted yet (still asks for “/mnt/data/haijing/simDeconv/Raw/GSE51984/RSEM_quant/” when it should be user-independent); 2) Please enlarge the section "Execution order" on the landing page of Github mentioning that each subfolder contains "XX_README.md" files with order instructions"; 3) Please rename Libraries.txt to Libraries.R and source it at the beginning of every script to avoid issues with library dependencies.

**Authors Response**

**Point-by-point responses to the reviewers’ comments:**

*We thank reviewer 2’s constructive comment and suggestions. In this response, we quoted reviewer 2’s comments in italic font and responded in regular black font.*

*Some major changes in the revised Manuscript:*

*Revised sections based on reviewer 2’s comment have been highlighted in yellow color.*

*We changed our Figure 1 and added one new Supplementary Figure (Supp Figure 15)*

Reviews & Responses

Based on their answer to my first primary comment from the first round of peer-review, Line 14 from the abstract (“due to lack of comprehensive benchmarks”) should be removed because it is not true. Moreover, lines 38-39 (“tested on inconsistent frameworks”) still should be further explained.

*Response: We deleted the “due to lack of comprehensive benchmarks” in the Abstract section (Manuscript: Line 13 - 16). We added the explanation of “inconsistent frameworks” in the Introduction section (Manuscript: Line 41 - 43).*

Their answer to my previous question “Were all these considered as individual cell types to make mixtures?” was not included anywhere in the manuscript (“We avoided using

heterogeneous samples, typically named by ‘whole blood’, ‘whole white blood’, or ‘pbmc’, for reference generation”).

*Response: We have now added the explanation of simulated mixture source in the Methods section (Manuscript: Line 474 - 478).*

Line 122 (“impact of RNA-seq quantification is trivial”) still misses the information provided by the authors in one of their answers “The library size normalization has trivial impacts on the Sim1_simModel because samples in Sim1_simModel have the same library size.” (they also observed it is not a trivial choice in their other simulations: lines 192-194).

*Response: We have now added a detailed explanation of quantification unit on Sim1_simModel and Sim1_libSize in the Results section (Manuscript: Line 197 – 200 and Line 209 – 215).*

Their new additions in grey throughout the manuscript require extensive English corrections (e.g. “Further studies are still in need decompose the influence of multicollinearity

issue and rare components” / “we explored the impacts of noise structure” / “More benchmarks derive accuracies of cell-type specific expression estimation is still in need” / “we relaxed the criteria by the controlling the percentages of sample number passing the expression threshold”… to name a few).

Response: We corrected the wordings, especially the Introduction and Discussion section.

On lines 532-534 authors have added “When a deconvolution returns NA values, we directly assign highest penalty for the evaluation metrics: r = -1, and mAD = 1.” Why would a deconvolution method return NA values? Which ones did? This is very relevant information that should be reported.

*Response: NA values can arise from two sources: deconvolution algorithm and correlation coefficient calculation.*

*Deconvolution method: We found in the Sim1 analysis, CAMfree (reference-free deconvolution method) will return NAs when it fails to converge. To compare the performances, we convert NAs to -1, the worst correlation outcome (Supplementary Figure 3) or 1, the worst Mean Absolute Deviance (Supplementary Figure 4). Based on our observation, CAMfree returns NAs when log-normal and normal noise models or high perturbation (P10) noise were used.*

*Correlation coefficient calculation: This type of NA values mainly occurred in Sim2 and Sim3, when we calculate cell-type specific accuracies, deconvolution methods like LinSeed and DeconRNASeq will return all-0-valued estimations across samples. Pearson’s correlation coefficient is computed based on the following equation:*

*Let’s assume that the vector x has all zero values, then the denominator turns to 0 which means the expression is not a legal fraction and thus the value is undefined.*

*We have now added the explanation in the Methods section (Line 597 - 605).*

Authors should still include in the manuscript their answer “While (we agree that) the weight matrix is not controllable by the user, researchers can roughly speculate some basic properties of the weight matrix from previous studies” when stating it as the most important factor affecting the deconvolution: Lines 289-291, 392-393 and Line 499. This is a key message that needs specific context to be correctly understood.

*Response: We have now added this sentence in the revised manuscript (Line 221 - 226).*

Supplementary Table 4 (“Gradient of Cell Types” tab) has to be included in the main manuscript in order to grasp how Sim2 was done (e.g. inside section “Benchmarking framework construction” before “Weight simulations”) .

*Response: We have now changed the index of Supplementary Table 4 to the Table 1 so that it will be in the main manuscript (Line 619 - 621).*

Authors did implement 2 very relevant analyses I requested (a) cases with 5 to 10 components where n-1 cell types have all small proportions and only one (always the same cell type) is big; b) cases with 5 to 10 components with equal proportions across all cell types). However, these are neither included in the manuscript nor in Supplemental (or at least not in the PDF version I got, which was missing SFig 1, 2, 6, 8, 9 and 10).

*Response: The results of these two analyses have been added in the Results section (Line 291- 299) and Supplementary Figure 15.*

The reviewer supports your points on points 1 and 2. For the third point, he/she feels that both you and the reviewer are “right”, so would recommend that you clarify this in the manuscript. He/she feels that the section from your rebuttal should be included:

[...] the major purpose of this paper is to systematically study different technical and biological factors on deconvolution analysis. Complex biological tissue with several confounded impacted factors is not suitable for this type of systematic comparison. In other words, with a few biological samples, we can’t track what factors are in play, and no clear guidance can be provided for future improvement of this technique. This is why systematic comparisons of deconvolution methods rely on in silico benchmark data4. The same logic also applies for scRNA-seq analysis; although a large number of scRNA-seq data is already available, researchers still need to design specific benchmarks for systematic comparisons.

3rd point from reviewer 1

“3) "When a deconvolution method returned NA value, we assign a penalty value (correlation: -1 and mAD: 1) to ensure the failure is reflective in the final evaluation metrics (Method section, line 532-534)". Does this mean that simply because a method does not incorporate a given cell type, it gets penalized? This is an important issue, because it could very well be that some methods perform badly simply because they do not incroprate some of the cell types that have been a priori selected by the authors (and this choice is indeed questionable - for instance why separating Memory and Naive B cells, but not the subtypes of CD4 cells?). For instance, many methods do not include predictions for oesinophil, a relatively rare cell type, or a distinction between Naive an Memory B cells, and will therefore be strongly penalized. Also, I do not understand how values have been computed in Figure 4E, since it looks like all methods could be applied to all cell types, which is clearly not the case when using methods with default parameters. Or did the authors retrain the algorithms with new profiles, as some methods enable to include user-defined profiles. This is all very confusing, and I'm afraid the authors need to check more precisely how they used the different methods and provide better explanations in the method section.”

*Response:*

*We have added the paragraph (highlighted with red) in the Introduction section of revised manuscript (Line 69 - 78).*

*Both Reviewer 1 and reviewer 2 have concerns on NA values, we have now added the explanation of the NA values in our Methods section (Line - 597 - 605).*

*For the impact of some specific cell type, we add the discussion on highly correlated cell types and rare cell component in our Results section (Line 281 - 290).*

*In Figure 4E, all methods were applied to all cell types. All eleven deconvolution methods tested in this manuscript support a user-defined reference as an input. To make the comparisons fair, in one benchmarking framework, we provided the same reference profile to all methods. We have clarified it in our Methods section (Line 462 - 466).*

Github:

Comments to the Github repository:* Authors have also improved their Github repository and provided data from a Google drive link. However: 1) this is not ideal for long-term storage so please upload it to Figshare or similar. 2) the code hasn’t been fully adapted yet (still asks for “/mnt/data/haijing/simDeconv/Raw/GSE51984/RSEM_quant/” when it should be user-independent); 23) Please enlarge the section “Execution order” on the landing page of Github mentioning that each subfolder contains “XX_README.md” files with order instructions”; 34) Please rename Libraries.txt to Libraries.R and source it at the beginning of every script to avoid issues with library dependencies.

*Response:*

*We have now uploaded raw & processed data to the Figshare and attached the Figshare link in our Github page. (Figshare link: https://figshare.com/projects/Data_for_the_manuscript_A_benchmark_for_RNA-seq_deconvolution_analysis_under_dynamic_testing_environments_/96908)*

*We have now changed the absolute path to the relative path in the script ./code/s1_preprocess/preprocess_GSE51984.R*

*We have now added the explanation in the Execution Order section.*

*We have now changed the format of the Libraries.txt file to Libraries.R and added explanation in the Github README.md file.*

**Third round of review**

**Reviewer 2**

The authors have answered all my comments satisfactorily and implemented the changes throughout the manuscript. The only remaining thing to do is changing "path = "/mnt/data/haijing/simDeconv/Raw/" to "path = "./Raw" in their three scripts ending with "_params_set.R" (lines 25, 28 and 29 respectively).

**Authors’ Response**

*We have now fixed the path issue in our source code.*
